# Extensive conservation of the proneuropeptide and peptide prohormone complement in mollusks

**DOI:** 10.1038/s41598-019-40949-0

**Published:** 2019-03-19

**Authors:** A. L. De Oliveira, A. Calcino, A. Wanninger

**Affiliations:** Department of Integrative Zoology, Faculty of Life Sciences, University of Vienna, Althanstraße 14, Vienna, 1090 Austria

## Abstract

As one of the most diverse groups of invertebrate animals, mollusks represent powerful models for neurobiological and developmental studies. Neuropeptides and peptide hormones are a heterogeneous class of signalling molecules involved in chemical communication between neurons and in neuroendocrine regulation. Here we present a fine-grained view of the molluscan neuropeptide and peptide hormone toolkit. Our results expand the distribution of several peptide families (e.g., prokineticin, insulin-related peptides, prohormone-4, LFRFamide) within Lophotrochozoa and provide evidence for an early origin of others (e.g., GNXQN/prohormone-2, neuroparsin). We identified a new peptide family broadly distributed among conchiferan mollusks, the PXRX family. We found the Wnt antagonist *dickkopf1/2/4* ortholog in lophotrochozoans and nematodes and reveal that the egg-laying hormone family is a DH44 homolog restricted to gastropods. Our data demonstrate that numerous peptides evolved much earlier than previously assumed and that key signalling elements are extensively conserved among extant mollusks.

## Introduction

Neuropeptides and peptide hormones constitute a heterogeneous group of evolutionarily related signalling protein molecules involved in neuro-modulation, neurotransduction, and hormonal functions^1^, that commonly act via G protein-couple receptors (GPCRs). Two major differences between neuropeptides and peptide hormones concern the biological system in which they are functional as well as their signalling targets. Neuropeptides are secreted by neuronal cells and act on neighboring targets (cell-cell contact) whereas peptide hormones diffuse over long distances via haemolymph or blood, affecting targets far from the signalling source. The latter mechanism is controlled by the endocrine system^2^.

Neuropeptides and peptide hormones are synthesized in the form of large inactive precursor molecules known as proneuropeptides (pNPs) or prohormones. They are redirected to the secretory apparatus and are further cleaved and modified to regulate homeostatic processes and distinct behaviours in animals^3^. Structurally, pNPs and prohormones share common characteristics such as the presence of an N-terminal signal peptide and one or more peptide sequences flanked by mono- or dibasic cleavage sites which are recognised by prohormone convertases. Each pNP and peptide prohormone may give rise to a single bioactive peptide, several copies of a single bioactive peptide, or more than one distinct bioactive peptide. Additional enzymatic processing steps, i.e. post-translational modifications (e.g., C-terminal alpha-amidation, N-terminal pyroglutamination) often occur before the generation of the active peptides^4–6^.

The recent improvement of DNA sequencing technologies accompanied by the substantial reduction of costs has expanded the investigation of neuropeptide and hormonal signalling systems beyond the classical model organisms such as the nematode *Caenorhabditis elegans*^7^, the fruit fly *Drosophila melanogaster*^8^, and human^9^. Thus, comparative research into the evolution and diversity of metazoan neuropeptides, peptide hormones, and their molecular components today involves a range of previously neglected taxa from virtually all major metazoan lineages.

Proneuropeptides are widespread in eumetazoans (all multicellular animals except sponges)^10,11^. The key components of the enzymatic toolkit essential for pNP and peptide prohormone processing, maturation, and secretion originated long before the emergence of Eumetazoa and are commonly recognized in organisms that lack a nervous system such as sponges and algae^12–14^. Within Lophotrochozoa (a major clade of bilaterally symmetrical protostome animals that includes groups as diverse as platyhelminths, annelids, mollusks, or brachiopods), comprehensive investigations of the neuropeptide and peptide hormonal signalling systems have been conducted in the annelids *Capitella teleta*^15^, *Helobdella robusta*^15^, and *Platynereis dumerilii*^16^, as well as in two platyhelminths, the parasitic *Schistosoma mansoni*^17^ and the free-living *Schmidtea mediterranea*^18^. The number of predicted peptide precursors (proneuropeptides and prohormones) in these species ranges from 13 in *S. mansoni* to 98 in *P. dumerilii*. These results demonstrate a tremendous variation in the composition of signalling peptides even in closely related organisms.

Mollusks comprise the most speciose and diverse lophotrochozoan phylum. They display highly variable behavioural and physiological repertoires, developmental pathways (ranging from indirect development via various larval types to direct development), and neuroanatomical features. Molluscan nervous systems vary widely in their degree of complexity. They may exhibit little or no anterior centralization and may lack ganglia along their four longitudinal nerve cords (e.g., in aculiferans and monoplacophorans)^19–23^ or may have multiple (pairs of) ganglia (e.g., in the majority of the conchiferan clades). Neural complexity in mollusks peaks in the highly centralized, lobular brains of cephalopods^24,25^. Despite these considerable morphological differences, thorough assessments of the diversity of proneuropeptides and peptide prohormones in mollusks are only available for a few individual gastropod^26–29^, bivalve^30,31^, and cephalopod species^32^ (Table 1). In the five remaining molluscan class-level taxa (Chaetodermomorpha, Neomeniomorpha, Polyplacophora, Scaphopoda, and Monoplacophora) comprehensive and systematic investigations that are focused on peptidergic signalling systems are still lacking.

**Table 1:** Summary of predicted peptide precursor genes identified in gastropod, bivalve, and cephalopod mollusks.

Organism	Class-level taxa	Data source	# of peptide precursors	References
*Lottia gigantea*	Gastropoda	Genome	67	^26^
*Theba pisana*		Transcriptome	35	^27^
*Deroceras reticulatum*		Transcriptome	65	^28^
*Charonia tritonis*		Transcriptome	60	^29^
*Pinctada fucata*	Bivalvia	Genome and transcriptome	31	^30^
*Crassostrea gigas*			44	
*Patinopecten yessoensis*			63	^31^
*Sepia officinalis*	Cephalopoda	Transcriptome	55	^32^

Previous studies have shown a high degree of conservation of the repertoire of neuropeptides and peptide hormones (e.g., achatin, allatotropin, elevenin, and LFRFamide) between gastropods, cephalopods, bivalves, and other phyla, corroborating the notion that these molecules originated early in animal evolution^10,11^. Screening molluscan databases for potential neuropeptides and peptide hormones resulted in the identification of hitherto unknown peptide families with representatives in other animal phyla such as annelids and insects^32^. Numerous peptide families that are restricted to Mollusca or individual molluscan class-level taxa were also identified^32–34^.

In order to elucidate the evolutionary history of peptide signalling molecules and to assess whether the complexity of neural systems is reflected in the diversity of proneuropeptide and peptide hormone complements in mollusks, we analysed 62 publicly available datasets covering 35 molluscan and 19 other metazoan species. Sequence data from Mollusca and nine other lophotrochozoan phyla were included: Annelida, Brachiopoda, Ectoprocta, Entoprocta, Gastrotricha, Nemertea, Phoronida, Platyhelminthes, and Rotifera. We identified 65 proneuropeptide and peptide prohormone families with homologs in one or more mollusk species. The homology of several other non-molluscan lophotrochozoan peptide sequences was confirmed and their relatedness with the molluscan pNP and peptide prohormones established (e.g., presence of shared conserved motifs, pattern of BLAST connections in the cluster maps). Our study represents the most complete and broad catalog of molluscan proneuropeptides and peptide hormones to date and constitutes an important resource for further investigations of molluscan and lophotrochozoan neural evolution, neurogenesis, and physiology.

## Results

### Prediction of molluscan and lophotrochozoan neuropeptidomes

Quality filtering of the molecular sequence databases followed by *de novo* assembly and identification of the coding sequence regions generated predicted protein datasets ranging from 12,808 (the shallow coverage of the chaetodermomorph *Chaetoderma* sp. data) to 606,184 sequences (combined ultra-deep *Dreissena rostriformis* libraries from different developmental stages). Assessments of completeness in the reconstructed protein datasets based on the presence of 978 benchmarking universal single copy metazoan orthologs^35^ showed a great variation ranging from 4.73% completeness in the basally branching protobranch bivalve *Yoldia limatula* to up to more than 90.0% in the brachiopod *Lingula anatina*, the scaphopod *Gadila tolmiei*, and the annelid *Capitella teleta*. The 454-sequenced libraries^36,37^ of the polyplacophoran *Chaetopleura apiculata*, the gastropods *Littorina littorea*, *Perotrochus lucaya*, and *Siphonaria pectinate*, the cephalopod *Nautilus pompilius*, and the bivalve *Yoldia limatula* present the highest number of missing BUSCOs. The BUSCO assessment results for the 54 lophotrochozoan proteomes are summarised in Supplementary Fig. S1. The established non-redundant lophotrochozoan neuropeptidomes (set of proneuropeptides and peptide prohormones) have between 173 (in the gastropod *Biomphalaria glabrata*) and 14,195 (combined *Dreissena rostriformis* transcriptomes) secreted protein sequences with all hallmarks of either a bona fide proneuropeptide or a peptide hormone (e.g., signal peptide, non-folded protein domain, and repetitive motif sites). The 54 lophotrochozoan non-redundant neuropeptidomes are mostly composed of full-length coding region protein sequences (Supplementary Dataset S1).

### The proneuropeptide/peptide prohormone complement of Mollusca

**Figure 1:**
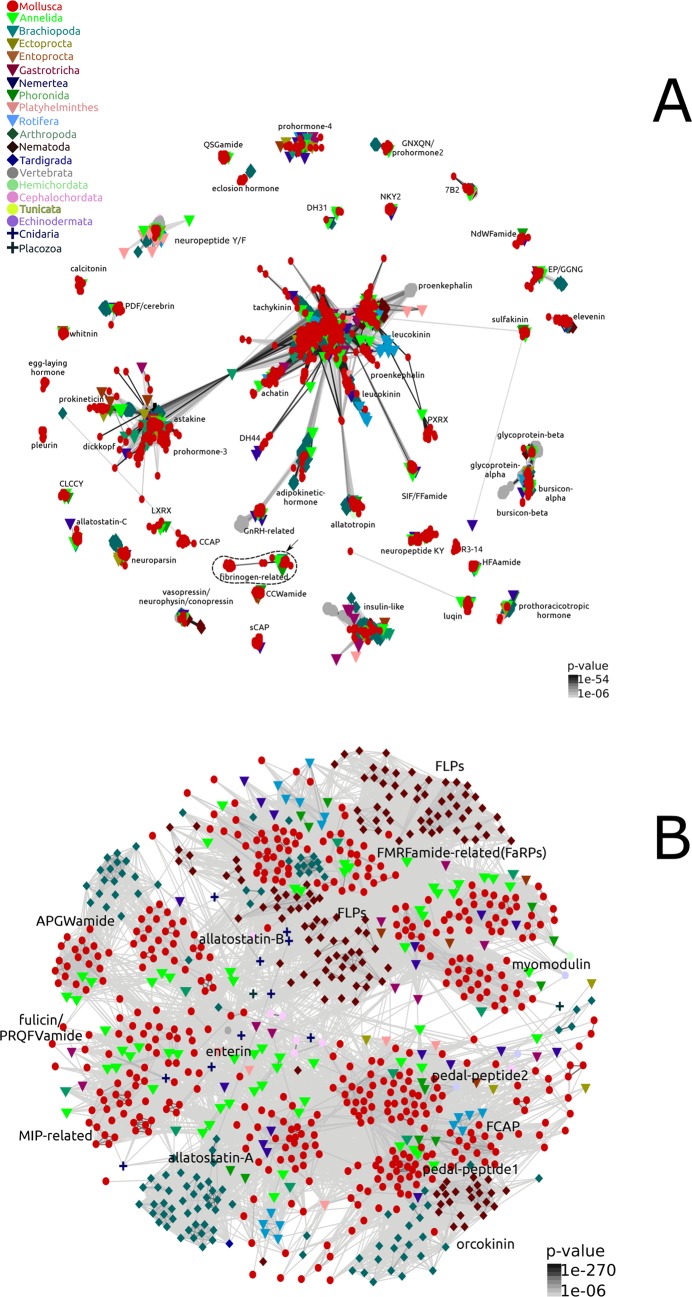
2D cluster maps of molluscan and lophotrochozoan peptide families. Color and shape of nodes are based on the different phyla used in the analysis. (**A**) Psi-blast 2D cluster map (3 iterations) of molluscan and lophotrochozoan peptide families. Edges correspond to psi-blast connections of P-value > 1e-06. (**B**) Non-iterative blastp 2D cluster map of repetitive peptide sequences. The central strongly connected cluster in the psi-blast 2D map (**A**) was reclustered with non-iterative blastp. The clusters were identified using convex-clustering, multiple sequence alignments, and motif identification. Edges correspond to blastp connections of P-value > 1e-06. The fibrinogen-related peptide cluster (marked with an arrow in (**A**)) indicates a false positive result (see Discussion for more details).

**Figure 2:**
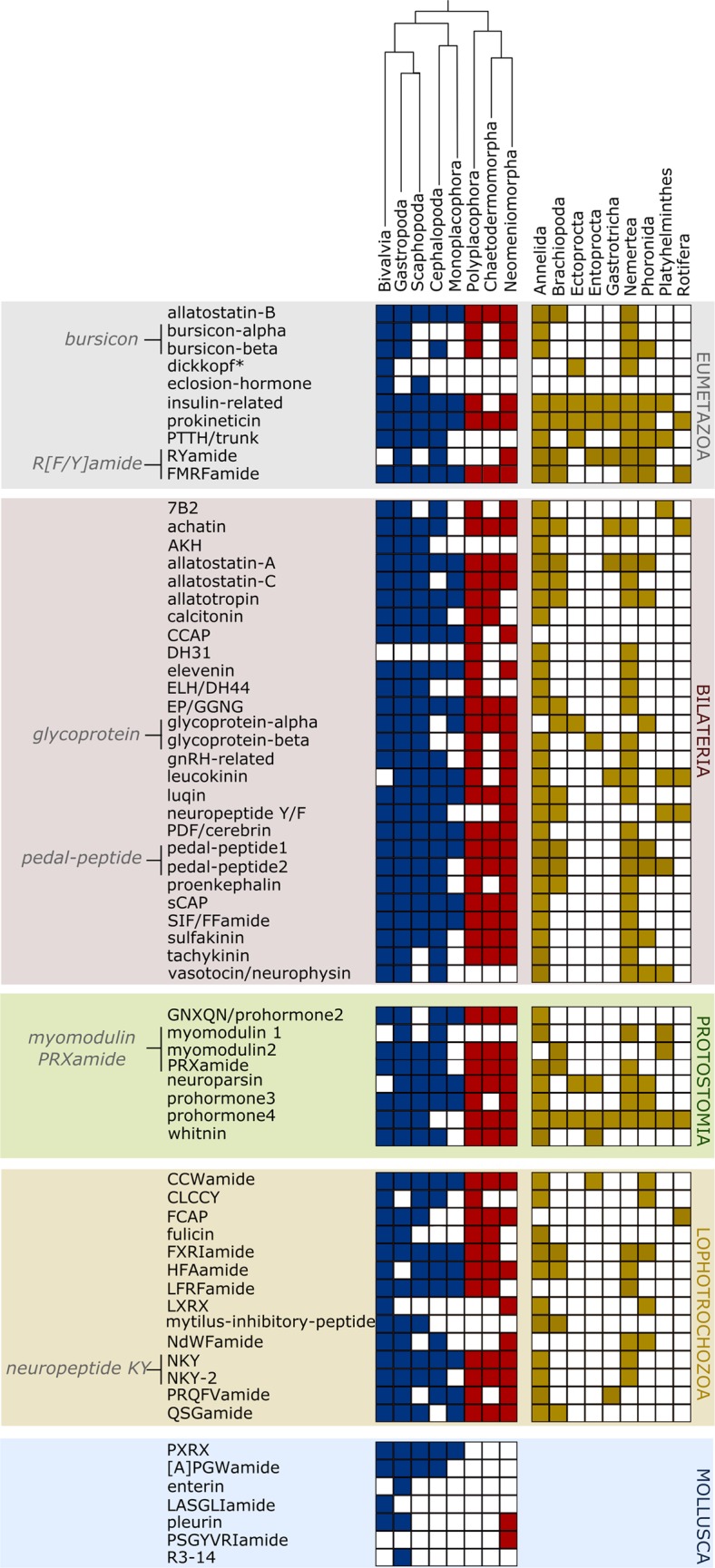
Minimum proneuropeptide/peptide prohormone complement of Mollusca. Peptide precursors were classified following criteria defined in^16^, distinguishing peptide families present in the last common ancestor (LCA) of eumetazoans, bilaterians, protostomians, and lophotrochozoans. Peptide families present in the LCA of Mollusca and different molluscan class-level taxa are also displayed. The differently coloured boxes correspond to the presence of a given peptide family in conchiferan (blue), aculiferan (red), and lophotrochozoan (yellow) representatives. Note that the dickkopf family proteins are not true neuropeptides or hormones, but rather secreted proteins that share a cysteine-rich domain with prokineticins.

**Figure 3:**
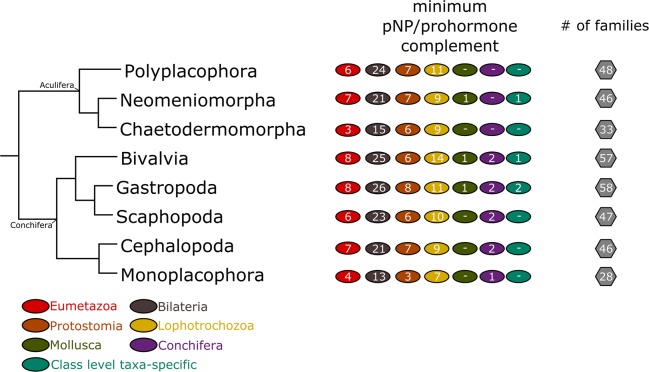
The distribution of the components of the pNP/peptide prohormone complement in molluscan class-level taxa using the currently widely accepted Conchifera/Aculifera hypothesis as a phylogenetic backbone. Coloured circles correspond to peptide families present in the last common ancestor of eumetazoans, bilaterians, protostomians, lophotrochozoans, mollusks, and, when present, conchiferans and specific class-level taxa, respectively. The numbers in the hexagons correspond to the minimum number of peptide families present in the last common ancestor of the various extant class-level taxa of Mollusca (in the right column).

Using a bioinformatic pipeline for proneuropeptide and peptide prohormonal identification adapted from previous surveys^10,16^, fine-grained 2D maps depicting the presence of major components of the molluscan neuropeptide/hormonal signalling systems were generated (Fig. 1; Supplementary Datasets S2 and S3). These depict hundreds of molluscan and lophotrochozoan homologs of known metazoan pNP/peptide prohormone families that were previously unknown from these clades. The deep molluscan taxonomic sampling identified 65 peptide families distributed in one or more molluscan taxa (Fig. 2). The minimum pNP/peptide prohormone complement of the eight class-level taxa of Mollusca ranges from 28 families in monoplacophorans to 58 in gastropods (Fig. 3). The majority of the proneuropeptide and peptide prohormone families found in mollusks were also identified in other lophotrochozoans such as annelids (51 families in common) and nemerteans (40 families in common) (Fig. 2). A full catalog of the mollusk/Lophotrochozoa-containing peptide families is provided in Supplementary Note S1.

### Eumetazoa-specific pNPs and peptide prohormones

Numerous peptide sequences retrieved from the molluscan and lophotrochozoan databases are also present in animals outside Lophotrochozoa, providing evidence that they were already present in the last common eumetazoan ancestor (Fig. 2). These include the cysteine-knot glycoprotein hormones bursicon-alpha and bursicon-beta, insulin-related peptides (IRPs), orthologs of the insect eclosion-hormone (EH), and the extracellular signalling molecule trunk (related to the arthropod prothoracicotropic hormone, PTTH). A variety of mature short peptides encoded by FMRFamide and RYamide pNPs were found in mollusks and seven of the nine lophotrochozoan phyla under investigation (Annelida, Brachiopoda, Entoprocta, Gastrotricha, Nemertea, Phoronida, and Rotifera; Fig. 2). Allatostatin-B or myoinhibitory peptides, characterised by the conserved N-terminal tryptophane residue (W) and the C-terminal Wamide motif in the bioactive pNP, were identified in all eight molluscan classes, annelids, brachiopods, and nemerteans.

**Figure 4:**
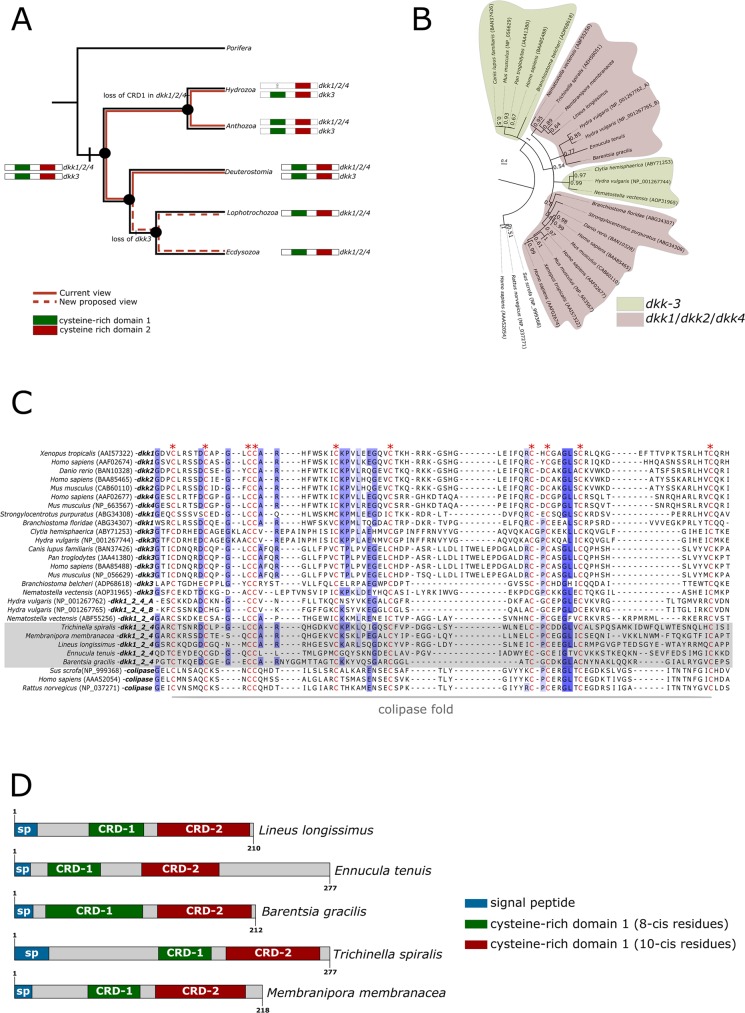
Evolution and distribution of dickkopf (dkk) proteins in Metazoa. (**A**) Traditional (red line) and novel (dotted red line) view of evolution and distribution of the *dkk 1/2/4 and dkk 3* orthologs, highlighting the presence of *dkk 1/2/4* orthologs in protostomian animals. (**B**) Bayesian phylogenetic analysis of dkk proteins using the cysteine-rich domain-2 (colipase fold) found in lophotrochozoan and ecdysozoan representatives. Sequences highlighted in bold correspond to the protostomian orthologs found in this study. NCBI accession numbers, when available, are displayed after the species names. The newly described dkk sequences are available in Additional file 3. Branch support values correspond to posterior probability values. Human, pig, and rat colipases were used as outgroups. (**C**) Multiple sequence alignment of the cysteine-rich domain-2 (colipase fold) showing the ten conserved cysteine residues as well as other conserved motifs in cnidarian, protostome, and deuterostome representatives. Lophotrochozoan and ecdysozoan orthologs are highlighted in the light gray box. (**D**) Domain structure of protostomian dkk sequences. Blue, green, and red boxes correspond to the signal peptide, cysteine-rich domain-1 (dkk domain), and cysteine-rich domain-2 (colipase fold), respectively.

One surprising outcome was the identification of *dickkopf1/2/4* orthologs, secreted proteins that contains cysteine-rich domains present in prokineticins and colipases, in ecdysozoan and lophotrochozoan representatives, expanding the phyletic distribution of this gene family to the entire Protostomia clade (Fig. 4A,B). All newly identified protostomian dickkopf sequences (dkk) contain a signal peptide and two conserved cysteine-rich domains (CRD-1 and CRD-2) in which the N-terminal domain (CRD-1) corresponds to the dickkopf domain *per se* and the C-terminal domain (CRD-2) corresponds to the colipase fold (Fig. 4A,C). Multiple sequence alignment revealed that the CRD-1 domains of the anthozoan cnidarian *Nematostella vectensis* and the protostomes all share eight cysteine residues. *Hydra dkk1/2/4* orthologs lack the CRD-1 domain (Fig. 4A). Multiple sequence alignment of the colipase CRD-2 domains shows that all protostome, cnidarian, and deuterostome sequences possess ten highly conserved cysteine residues (Fig. 4C). Outside the shared cysteine residues of the two CRDs, the dkk proteins show little sequence similarity. Bayesian phylogenetic inferences performed with CRD-2 domains recovered five distinct well-supported dkk clusters, two corresponding to the dkk-3 family (one belonging to deuterostomes and the other one to cnidarians) and the remaining three to the dkk1/2/4 family (Fig. 4B). The parasitic nematode *Trichinella spirales*, the ectoproct *Membranipora membranacea*, and the nemertean *Lineus longissimus* sequences are closely related to the *Nematostella dkk/1/2/4* ortholog, while the remaining two lophotrochozoans, the bivalve mollusk *Ennucula tenuis* and the entoproct *Barentsia gracilis*, are more closely related to the hydrozoan *Hydra vulgaris*. Although in-cluster resolution was robust, a lack of resolution between clusters prevented a phylogenetic classification of two dkk-3 and the three dkk/1/2/4 groups relative to each other.

Another pNP family with a C-terminal colipase fold-related domain, named prokineticin, was identified in virtually all lophotrochozoan phyla sampled, with the exception of Platyhelminthes (Fig. 2). Thirty-five transcripts belonging to the monoplacophoran *Laevipilina hyalina* with homology to other metazoan prokineticins were found (Supplementary Dataset S2). Multiple sequence alignment and phylogenetic analyses show the presence of four groups of prokineticin-like peptides with high posterior probability support values in *Laevipilina* (Supplementary Fig. S2).

### Bilateria-specific pNPs and peptide prohormones

Numerous pNP/hormone representatives found in mollusks are present in the vast majority of other bilaterian clades, including 7B2, achatin, allatotropin, adipokinetic-hormone (AKH), allatostatin-C, crustacean cardio-active peptide (CCAP), elevenin (L11), glycoprotein-alpha and glycoprotein-beta, gonadotropin-releasing hormone (GnRH), leucokinin, neuropeptide Y/F, proenkephalin, sulfakinin, tachykinin, and vasotocin/neurophysin (Fig. 2). In many instances, peptide families were identified in all eight class-level taxa of Mollusca, such as EP, SIF/FFamide, allatostatin-A, luqin, pigment dispersing factor (PDF), pedal-peptide (ortholog of the ecdyszoan orcokinin), and small cardioactive peptide (sCAP). The insect single copy PDF pNPs formed a well-connected cluster with gastropod cerebrins and a number of other, previously uncharacterised, molluscan, annelid, and nemertean pNPs.

Despite orthology of calcitonin and diuretic hormone 31 (DH31), these two pNP families are split into two distinct clusters on the 2D cluster map (Fig. 1A). The calcitonin pNP is present in aculiferan and conchiferan representatives, and, apart from the truncated *Ennucula tenuis* sequence, all sequences contain the two conserved cysteine residues in the mature neuropeptide. The polyplacophoran *Leptochiton rugatus* is the only investigated mollusk with both calcitonin and DH31 orthologs (Supplementary Note S1). As in the *Platynereis dumerilii* and the insect DH31 orthologs, the molluscan DH31 sequence lacks cysteine residues in the bioactive peptide domain (Supplementary Note S1).

**Figure 5:**
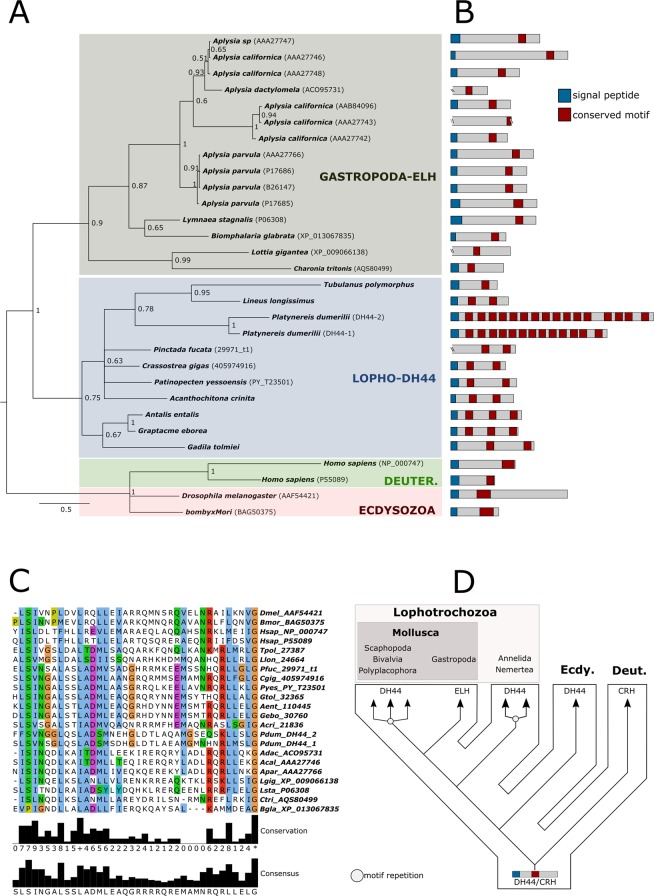
Evolution of DH44-ELH peptide hormone families in lophotrochozoans, ecdysozoans, and deuterostomes. (**A**) Bayesian phylogenetic analysis using trimmed DH44/ELH protostomian sequences. Note the presence of three well-supported clusters: lophotrochozoan DH44, gastropod ELH, and ecdysozoan/deuterostome DH44/CRH. NCBI accession numbers, when available, are displayed after the species names. The newly described DH44/ELH sequences are available in Additional file 3. Branch support values correspond to posterior probability values. (**B**) Domain structure of DH44 and ELH sequences showing the signal peptide (blue box) and shared conserved motifs corresponding to the predicted amidated peptides (red boxes). (**C**) Multiple sequence alignment of ELH, DH44, and CRH bioactive domains in metazoans. Species names are abbreviated for convenience (e.g., *Drosophila melanogaster* = Dmel; *Bombyx mori* = Bmor). The conservation histogram corresponds to the number of conserved amino acid physico-chemical properties for each column of the alignment. The consensus displayed below the alignment is the percentage of the modal residue per column including gaps. (**D**) New evolutionary scenario of ELH/DH44 prohormone sequences within Mollusca. White circles correspond to the presence of motif repetitions within the precursor sequences.

As with calcitonin/DH31, the orthologous egg laying hormone (ELH) and DH44 families are split into two distinct clusters (Fig. 1A). Identification of the conserved ELH/DH44 motif in annelids, nemerteans, mollusks, and arthropods showed different patterns of peptide repetition duplications, ranging from one motif in flies (*Drosophila melanogaster*), silkworm (*Bombyx mori*), and the nemertean *Tubulanus polymorphus*, to up to 16 in the polychaete annelid *Platynereis dumerilii* (Fig. 5B, Supplementary Note S2). Within Mollusca, dentaliid scaphopods (*Graptacme eborea* and *Antalis entalis)* harbor three repetitions of the motif, whereas the gadiliid scaphopod *Gadila tolmiei*, the bivalves *Pinctada fucata, Crassostrea gigas*, and *Patinopecten yessoensis*, as well the polyplacophoran *Acanchochitona crinita* only have two (Fig. 5B, Supplementary Note S2). Multiple sequence alignments using DH44/ELH and corticotropin-releasing bioactive hormone domains showed higher conservation of amino acid positions within the C- and N- terminal regions (Fig. 5C). Bayesian phylogenetic inferences using molluscan ELH sequences and the protostomian diuretic hormone 44 (DH44) as well as the deuterostome corticotropin-releasing factor (CRH) orthologs revealed the presence of three well-supported and distinct clades (Fig. 5A). The first contains the ecdysozoan and deuterostome sequences, the second is exclusively composed of gastropod ELH sequences, and the third comprises the remaining non-gastropod mollusk, the annelid, and the nemertean sequences (Fig. 5A). Thereby, the bivalve, scaphopod, and polyplacophoran peptide sequences show a higher degree of similarity to the *Platynereis* DH44 and the nemertean sequences than to their closest gastropod relatives (Fig. 5A). These results are consistent with estimates of evolutionary divergence, which show that the sequences of the bivalves *C. gigas*, *P. fucata*, *P. yessoensis*, the polyplacophoran *A. crinita*, and the scaphopod *G. tolmiei* are less divergent from the annelid and nemertean sequences than from their gastropod counterparts (Supplementary Dataset S4).

### Protostomia-specific pNPs and peptide prohormones

Eight molluscan pNP/peptide prohormone families originated in the stem protostome (Fig. 2), including prohormone-3 and prohormone-4, two myomodulin proneuropeptide precursors, neuroparsin, and PKYMDT/whitnin. Lophotrochozoan myomodulin pNPs generally yield multiple copies of small LRL- and LRMamide bioactive peptides, with the conserved motif located at the C-terminal end (although variations were observed in conchiferan and nemertean representatives, e.g., VRL-, LRV-, and VRMamide) (Additional figure 9). Conversely, the sequence composition of the N-terminal region of the bioactive myomodulin neuropeptides is highly variable, resulting in the production of numerous distinct peptides from each precursor neuropeptide. In the case of the aplacophoran mollusks, each bioactive peptide produced from the myomodulin-2 pNPs is unique, while in gastropod and cephalopod myomodulin-1 pNPs multiple identical copies of the bioactive peptides are present (Supplementary Note S3). In addition to LRL- and LRMamide peptides, molluscan and lophotrochozoan myomodulin pNPs (with the exception of gastropod myomodulin-1 and platyhelminth pNPs) encode a distinct class of PRXamide bioactive peptides (see Supplementary Note S3).

The GNXQN family grouped together with the insect prohormone-2 peptides and forms a well-resolved cluster (Fig. 1A). Motif searches revealed the presence of a highly conserved region (GN[QHR]QN) shared among all protostomians towards the N-terminal of all GNXQN/prohormone-2 pNPs (Supplementary Note S1).

### Lophotrochozoa-specific pNPs and peptide prohormones

Fourteen lophotrochozoan-specific peptide families were identified (Fig. 2). These include families previously restricted to individual molluscan classes (i.e. Gastropoda and/or Bivalvia) such as the four repetitive peptide families LFRFamide, PRQFVamide, feeding circuit-activating peptide (FCAP), Mytilus inhibitory peptides (MIP), and the D-amino acid-containing peptide family NdWFamide.

Precursors of the lophotrochozoan neuropeptide KY (NKY) form two distinct well-defined clusters of divergent proneuropeptide subgroups, NKY-1 and NKY-2. NKY family members are present in all eight class-level taxa of Mollusca, as well as in annelids and nemerteans. Multiple sequence alignments confirmed the presence of the conserved diagnostic lysine (Lys:K) and tyrosine (Tyr;Y) residues at the N- and C-terminal ends of these sequences. Conversely, the central region of the two NKY precursors, NKY-1 and NKY-2, differ considerably, being represented by FW[RQ]P[LM]G[YG] and G[YF]WIWMPAQG consensus peptide sequences, respectively.

The feeding circuit-activating peptide (FCAP) was identified in six of the eight molluscan class-level taxa and in all rotiferan taxa analysed here (*Rotaria tardigrada, Rotaria socialis*, and *Rotaria sordida*) (Fig. 2; Supplementary Note S4). The molluscan pNPs contain multiple FCAP copies ranging from six in the pulmonate slug *Deroceras reticulatum* to up to 28 in the limpet *Lottia gigantea* (Supplementary Note S4). The molluscan FCAP-bioactive peptides are usually 13 amino acids long; however, differences in their length were observed (Supplementary Note S4). The rotiferan FCAP-related bioactive peptides are shorter (with a fixed length of 11 amino acids) than their molluscan counterparts and are present in eight copies in the rotiferans *R. sordida* and *R. socialis* and in nine copies in *R. tardigrada* (Supplementary Note S4). All lophotrochozoan FCAP-bioactive peptides are composed of related sequences that show species-specific variability towards the N-terminal region (Supplementary Note S4).

### Mollusca-specific pNPs and peptide prohormones

Seven peptide families with a distribution restricted to mollusks were recovered in the analysis (Fig. 2). These include two well-known gastropod peptide families, abdominal ganglion (R3–14) and enterin, while the more widely distributed [A]PGWamides were found in all conchiferans except Monoplacophora. Pleurins were recovered from bivalves, gastropods, and neomeniomorphs. Two pNP families composed of short potential bioactive peptides, referred to as LASGLI- and PSGYVRIamide, were identified in the bivalve *Dreissena rostriformis* as well as in the aplacophorans *Wirenia argentea* and *Gymnomenia pellucida*. A small peripheral group connected to the central cluster (Fig. 1B) composed solely of conchiferan pNP sequences was recovered (Fig. 2). The members of this peptide family showed no significant similarity against any known neuropeptide sequences available in the nr-database and thus likely represent an independent and divergent pNP family that evolved from sequences present in the central cluster (Fig. 1A). All the sequences in this pNP family possess four conserved cysteine residues that are likely to give rise to two intramolecular disulfide bridges (Supplementary Note S1). They also possess a conserved P[FM]R[WY] protein motif, with the exception of two sequences belonging to the bivalve *Dreissena rostriformis*. In accordance with conventions for pNP annotation, we name this conchiferan pNP family PXRX.

## Discussion

### Development of an *in silico* pipeline for proneuropeptide and peptide prohormone identification in Lophotrochozoa

**Figure 6:**
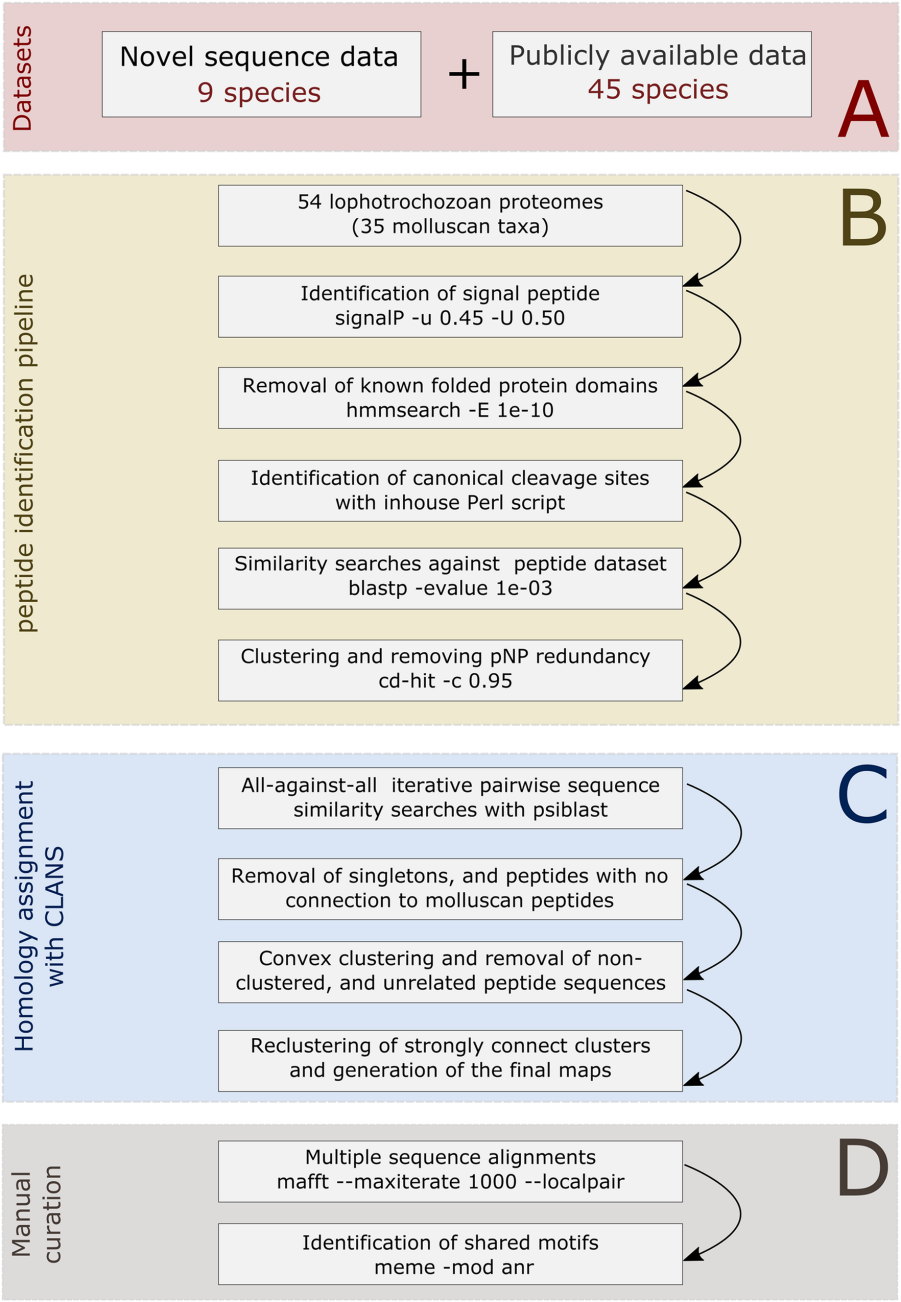
Bioinformatic pipeline developed for the identification and classification of molluscan and lophotrochozoan pNP and prohormone sequences. (**A**) The lophotrochozoan and molluscan databases were downloaded from Sequence read archive (https://www.ncbi.nlm.nih.gov/sra), pre-processed, and assembled locally (not shown). (**B**) Predicted coding sequence regions from genomic data were downloaded and used when available. Identification of the *sine-qua-non* prerequisites present in the peptide sequences, clustering, and removal of false positive sequences were performed with several bioinformatics tools (e.g., signal, blast, hmmsearch). (**C**) Orthology assignments and phylogenetic analysis were performed using all-against-all comparisons with psi-blast and blastp as implemented in the CLANS software. (**D**) Downstream annotation of the identified peptide sequences were aided through multiple sequence alignments, motif identification, and manual inspection.

No single best method has yet been established for the identification and retrieval of pNP and peptide prohormone sequences from genomic or transcriptomic databases. In 2013, two independent studies laid the framework for large-scale pNP and prohormone identification in metazoans^10,11^ and subsequent studies have employed modified versions of these *in silico* pipelines^16,32^. Herein, we present an updated bioinformatic pipeline for pNP and peptide hormone identification and annotation (Fig. 6) which has resulted in the identification and phylogenetic classification of hundreds of new pNPs and peptide hormones. Our greatly expanded but conservative new estimates of the pNP and peptide hormone complements of the eight molluscan class-level taxa are testament to the robustness of this pipeline.

It is difficult to state decisively that any particular peptide family is absent from the molecular databases analysed in our study. Methodological biases introduced during the data production and assembly steps, in addition to the meticulous avoidance of false positives, dictated by the stringency of the parameter settings used by the bioinformatics tools in the pipeline (e.g., signalP, hmmsearch, blastp), may have hindered the identification of some sequences. Some issues with the identification of molluscan and lophotrochozoan FMRFamide precursors may serve as example. FMRFamide peptides (Phe-Met-Arg-Phe-NH2) constitute one of the most well-known neuropeptide families studied in Mollusca since their discovery as a cardioacceleratory peptide^38^. They have since been identified in seven of the eight class-level taxa of mollusks^22,39,40^, with the exception of Monoplacophora. However, the pipeline described herein failed to retrieve these sequences from the investigated databases. A careful inspection showed that FMRFamide pNP sequences belonging to all eight molluscan class-level taxa and other lophotrochozoan phyla (Brachiopoda and Nemertea), were later filtered out during the hmmsearch step in which those sequences with matches to any member of either the PfamA or PfamB database were removed (Fig. 6B: “Removal of known folded protein domains”). This specific step was added in the pipeline in order to remove non-neuropeptide folded protein domain-containing sequences (with few exceptions, e.g., insulin-like domains). Manual curation of the resulting candidates revealed that matches against the pfamB model PF01581 (“FMRFamide-related peptide family”) had removed FMRFamide-related peptides from this list.

Our analysis revealed an erroneous protein annotation in a previous study^10^. A cluster of peptides from various lophotrochozoans (mollusks, phoronids, brachiopods, and annelids) and one ecdysozoan, the scorpion *Mesobuthus gibbosus*, was identified in the analysis (Fig. 1A). All these sequences, except those of the gastropod limpets *Lottia goshimai* and *Lottia gigantea*, share a conserved fibrinogen-related domain (FReD) towards the C-terminal end of the proteins. These proteins could easily be misinterpreted as a novel protostomian peptide family^10^; however a more thorough investigation revealed that this family is not composed of neuropeptide or peptide hormone sequences, but rather of secreted proteins with a globular fibrinogen domain. This transitive annotation error was caused by a spurious match that evaded detection by various quality filters used in the previous study^10^ (G. Jékely, personal communication, January, 2019), and again demonstrates the importance of manual curation in highly automated methods.

It is important to note that the aforementioned limitations are not solely restricted to this particular work but are also present in other studies concerning pNP and peptide hormone identification. To elucidate the complete pNP and peptide prohormone repertoire of metazoans, rigorous manual inspection, and techniques such as mass-spectrometry, represent powerful tools to complement *in silico* automated bioinformatic screenings^16,18,32,41–43^. Additionally, as suggested by Veenstra^26^, the identification and characterisation of G protein-coupled receptors (GPCRs) is a useful approach to fully understand the evolutionary history of a given peptide family, given the long-term coevolution of receptor-ligand pairs^10,11,44,45^.

### Mollusks as important models for clarifying the evolution and diversification of neuropeptide and peptide hormone families in metazoans

Studies focusing on the lophotrochozoan proneuropeptide and peptide hormone complement are still restricted to a few mollusks^26–32^, flatworms^18^, and annelids^15,16^. To fill this gap of knowledge, molecular databases for the different eight class-level taxa of Mollusca as well as nine major additional lophotrochozoan phyla were mined for the presence of pNP and prohormone sequences.

The wide taxon sampling spanning the extant diversity of lophotrochozoan phyla showed that many peptide families that had previously only been known from annelids and mollusks (e.g. NKY, FXRI, LXRX, CLCCY)^16^ have orthologs in other lophotrochozoan phyla, rendering them *bona fide* lophotrochozoan families (i.e. peptide families that emerged at the base of Lophotrochozoa). Moreover, peptide families that were hitherto only known from mollusks are shown here to be widespread in other lophotrochozoans, such as the LFRFamide, PRQFVamide, NdWFamide, feeding circuit-activating peptide (FCAP), and Mytilus inhibitory peptide (MIP) families (Fig. 2).

A few gene expression studies involving two of the aforementioned families, MIP and LFRFamide, have been performed in conchiferan mollusks^46–51^. Comparative physiological investigations involving MIPs in the bivalves *Mytilus edulis* and *Meretrix lusoria*^46^ as well as in the gastropods *Achatina fulica, Aplysia californica*, and *Aplysia kurodai*^48^ showed a strong inhibitory impact of these peptides on the contraction of different muscles in these animals. Regarding the LFRFamide peptides, a different scenario was revealed. In gastropods, LFRFamide peptides had an inhibitory activity on F2 neurons^47^ as well as on the control of the feeding and reproduction behaviour in the snail *Lymnaea stagnalis* during schistosomiasis infections^49^. In the oyster *Crassostrea gigas*^51^ and in the squid *Sepia officinalis*^50^ LFRFamide peptides are involved in energy metabolism and in the tonus and amplitude of rectal contraction.

Taken together, our results point to a pNP and peptide prohormone repertoire with evolutionary origins in Lophotrochozoa that consists of a minimum of 15 families, thus expanding the complement of ten previously identified families^16^. This finding, in combination with gene expression and functional studies, will enable testing of putative functions of these peptides in a broad range of lophotrochozoan taxa.

As a result of different evolutionary constraints^52,53^ and patterns of domain repetition and sequence divergence (i.e. little sequence similarity shared by related peptides from different phyla), the clustering approach is a robust method to elucidate and propose new evolutionary scenarios for pNPs and hormones. In addition, traditional phylogenetic reconstruction methods (e.g., maximum likelihood, Bayesian) become prohibitive and prone to errors when thousands of sequences are simultaneously analysed^54^. The establishment of a close evolutionary relationship between insect prohormone-2 and the lophotrochozoan GNXQN family exemplifies how this method can expose the interconnectedness of peptide families that were previously unknown to be related. Prohormone-2 was fully characterised by Hummon *et al*.^55^ as a NVPIYQEPRF-containing neuropeptide in many insects, whereas GNXQN pNPs were first described in annelids, bivalves, and gastropods^16^. Our analysis not only expands the known phylogenetic distribution of the GNXQN pNPs to the remaining molluscan class-level taxa, with the exception of Scaphopoda, but also indicates a homologous relationship between these two families. This points to a deeper origin of these families back to the last common protostomian ancestor.

A closer look into the prohormone-2/GNXQN cluster shows that insect prohormones are directly linked to some molluscan sequences. Likewise, the annelid sequences are also linked to the mollusks; however, no direct link exists between the annelids and insects. Had such an analysis been conducted without the inclusion of the molluscan prohormone-2/GNXQN orthologs, no link would have been observed to indicate a relationship between prohormone-2 in insects and GNXQN in annelids (Fig. 1A; Supplementary Dataset S2). This result exemplifies the importance of broad taxonomic sampling when annotating pNPs and peptide hormones.

Our analysis revealed the presence of dickkopf (dkk) sequences in lophotrochozoan and ecdysozoan representatives, which had hitherto been considered lost in the protostome lineage^56^. Dkks constitute a family that plays an important and ancient role in animal development by antagonising canonical Wnt signalling by competing with the Wnt-Frizzled complex for binding to the LRP receptors^57–60^. Despite not classified as a neuropeptide or peptide hormones *per se*, dkk share a cysteine-rich domain present in prokineticins and colipases^10,61^. Our analysis indicates the presence of two dkk genes in the last common ancestor of cnidarians and bilaterians, *dkk1/2/4* and *dkk3*, in which the first gave rise to the vertebrate *dkk1, dkk2*, and *dkk4* paralogs via gene duplication^62^. *In silico* data mining of genomic and transcriptomic databases of model organisms, such as *Drosophila melanogaster* and *Caenorhabditis elegans*, have so far failed to recover any dkk orthologs within Protostomia^56^. However, we found ecdysozoan and lophotrochozoan *dkk1/2/4* orthologs retrieved from nematodes, mollusks, ectoprocts, entoprocts, and nemerteans, which contain the two diagnostic cysteine-rich domains. These results demonstrate that the *dkk 1/2/4* ortholog was already present in the last common protostomian ancestor, while its paralog *dkk3* was secondarily lost in ecdysozoans and lophotrochozoans. Whether or not the Wnt-Dickkopf antagonism was functionally maintained in Ecdysozoa and Lophotrochozoa is yet to be demonstrated.

Since its discovery and isolation from the marine gastropod *Aplysia californica*^63,64^, the egg-laying hormone (ELH) has been subject to a number of studies focused on the molecular and neurophysiological mechanisms that dictate complex animal behaviour. When released into the hemocoel of a sexually mature gastropod, a series of behaviours are triggered (e.g., cessation of locomotion, inhibition of feeding, head movements), resulting in the extrusion of the egg mass^65^. While ELH was initially only known from gastropod mollusks^26,66,67^, recent studies have confirmed its presence in many species of bivalves^30,68^. Furthermore, ELH has been shown to be a homolog of the deuterostome corticotropin-releasing hormone (CRH) and the ecdysozoan and lophotrochozoan diuretic hormone 44^11,16^.

Phylogenetic analyses using the bioactive ELH domains showed that all molluscan sequences formed a unique clade^30^. Our results, using the bioactive ELH/DH44/CRH domain and its N-terminal flanking region, show that all gastropod ELH sequences form an independent and lineage-specific clade as sister group to the remaining molluscan and lophotrochozoan DH44 sequences. Interestingly, no ELH/DH44 sequences were retrieved from any cephalopod databases, including the predicted proteins from the *Octopus bimaculoides* genome^69^. These results are in agreement with another recent study that failed to retrieve any ELH/DH44 orthologs in transcriptomes built from the central nervous system of cuttlefish sampled during spawning^32^. Furthermore, similarity searches using the genome of *Euprymna scolopes* confirmed this same scenario in the squid, pointing to a likely loss of the ELH/DH44 in the Cephalopoda lineage (H. Schmidbaur & O. Simakov, personal communication, May, 2018). It is difficult to assess whether this evolutionary scenario is underlain by changes in the role of these genes in annelids, nemerteans, and the different molluscan class-level taxa, since no comparative functional studies with DH44/ELH hormones have been reported outside of Gastropoda. In the ecdysozoan *Drosophila*, DH44 is involved in water regulation, excretion by the use of Malpighian tubules, and detection and consumption of nutritive sugars, but not in reproductive behaviour^70–72^. However, immunolocalization studies demonstrated that ELH-like peptides might play a role in the spawning processes of other ecdysozoans, e.g., decapod crustaceans^73,74^.

### Evolution of the neuropeptide and hormone complement within Mollusca

Mollusks show a huge diversity of body plans and nervous system complexity^24,75^. Consequently, proneuropeptide and peptide prohormone toolkits in different mollusks constitute a valuable resource to elucidate the molecular mechanisms that control their development, growth, reproduction, and physiology. Comparative studies within Mollusca are still rare, and the few thorough analyses focusing on the pNP and prohormone complement are almost exclusively focused on individual gastropod and bivalve species^26–31^. Our work provides the repertoire of pNP and peptide prohormone signalling molecules for all the eight extant class-level taxa of Mollusca, including the understudied aculiferans, monoplacophorans, and scaphopods. The minimum class-level pNP/prohormone complement ranges from 28 to up to 59 in conchiferans and from 33 to 49 in aculiferans. The analyses revealed an unexpected conservation in the toolkit of pNPs and hormones within the phylum, regardless of the complexity of the nervous system and life styles of the respective protagonists (e.g., highly mobile predators versus slowly moving or sessile filter-feeders). FMRFamide, allatostatin-A and -B, NKY, pedal-peptides, and luqin are families shared by all molluscan class-level taxa. Additionally, the peptide families retrieved from the molluscan databases show homology to virtually all described eumetazoan, bilaterian, protostomian, and lophotrochozoan families^10,11,16^ and only in a few cases lineage-specific innovations in the peptide complement were observed (Fig. 2).

In some cases, peptide families were restricted to a limited number of molluscan class-level taxa. This is the case for the dickkopf (Fig. 4) and DH31 families. Previous studies claimed the secondary loss of DH31 in mollusks and the loss of dickkopf in protostomes^16^. Our results, however, show the presence of DH31 in a polyplacophoran (Fig. 2; Supplementary Dataset S2 and Supplementary Note S1) and dickkopf in lophotrochozoans and at least one nematode (*Trichinella spiralis*). These findings demonstrate the importance of comparative analyses and broad taxon sampling in order to clarify the evolution of peptide families in metazoans.

Comparative studies suggest that regulatory gene families (i.e. protocadherins and C2H2s), post-transcriptional mechanisms (i.e. RNA-editing), genome rearrangements, and extensive transposable element activity are major forces behind the behavioural repertoire (e.g., camouflage displays, problem solving, and observational learning) and the complex central nervous system (CNS) in cephalopods^68,76^. Our analysis suggests that the evolution of the complex CNS and the sophisticated behavioural repertoire of cephalopods was not paralleled by lineage-specific expansions of pNP or peptide prohormone families. Although our homology-based approach for pNPs/peptide prohormone identification might have failed to identify particularly divergent homologs and lineage-specific peptide families, the low number of peptide families (38 in total) identified using mass-spectrometry on the CNS of cuttlefish (*Sepia officinalis*) further corroborates our conclusions^32^.

The neuropeptide/hormone complement described here shows considerable overlap with the results of previous works on gastropods, bivalves, and cephalopods. However, several peptide families (generally short amidated bioactive peptides) with either a broad (e.g., PXXXamide, Samide, and SPamide families^32^) or a highly restricted phyletic distribution (e.g., CCFRamide^16^), even down to the species level (e.g., the scallop-specific GNamide, LRYamide, and Vamide families^31^), were not recovered in our study. It is therefore important to stress that the peptide families recovered in our study must not be regarded exhaustive, but rather as the minimum peptide complement present in the major class-level taxa of Mollusca.

## Conclusions

The phylum Mollusca comprises more than 200,000 extant species and harbors a plethora of distinct body plans, neural architectures, and forms of behaviour. Through a comparative and integrative approach using *in silico* protocols and sequence similarity-based clustering, a detailed overview of the minimum proneuropeptide/hormone complement of all extant class-level taxa of Mollusca was obtained. Our study provides a high-quality, manually curated catalog containing multiple sequence alignments and peptide logos for 65 metazoan proneuropeptide/peptide prohormone families. We identified a conchiferan proneuropeptide/prohormone family (PXRX), expanded the phyletic distribution of others (e.g., neuroparsin, DH31), and established the homology of seemingly unrelated peptides (e.g., GNXQN and prohormone-2). We show for the first time the presence of a *dkk-1/2/4* ortholog gene in protostomes, whereby the lophotrochozoan and ecdysozoan sequences possess the two diagnostic cysteine-rich dickkopf and colipase domains. ELH peptides are lineage-specific to gastropods but are closely related to their lophotrochozoan and non-gastropod molluscan orthologs, the diuretic hormone 44. Our results suggest that the complex nervous system and the extraordinary behavioural repertoire of cephalopods are not correlated with innovations of the downstream signalling elements (i.e. neuropeptides and hormones). Our pioneering study provides an important stepping stone towards a better understanding of the function and evolution of these conserved peptides not only in mollusks, but also in a wide range of other metazoans.

## Material and Methods

### Data collection, filtering, sequence reconstruction, proteome prediction, and completeness assessment

In order to identify as many molluscan and lophotrochozoan peptide groups as possible, several transcriptomes belonging to different class-level taxa of Mollusca and other lophotrochozoan phyla were downloaded from Sequence Read Archive database (www.ncbi.nlm.nih.gov/sra) and combined with molluscan transcriptomes generated by our group as described earlier^77^. Predicted coding sequence regions from genomic data were downloaded and included when available. The summary concerning the species, phyla, SRA accession numbers, and the file transfer protocol addresses (FTP) of the molecular data used in this study are available in Supplementary Dataset S5.

The Illumina datasets retrieved from SRA were subject to a cleaning procedure (identification of adapters, poor quality regions) using trimmomatic^78^ and were reconstructed with IDBA-tran^79^ using the parameters –max_isoforms and -step defined as 1 and 5, respectively. The 454 databases were reconstructed using successive rounds of assembly with MIRA4 and CAP3 programs using default parameters^80,81^. The prediction of coding sequence regions from the reconstructed transcriptomes was performed with TransDecoder (http://transdecoder.github.io/) and only the longest coding sequence region of each reconstructed transcript was retained for the subsequent analyses (Fig. 6A). The completeness of the individual proteomes was assessed with BUSCO^35^ with the default parameters using the pre-defined 978 metazoan Benchmarking set of Universal Single-Copy Orthologs. The proteomes were classified into BUSCO metrics as follows: complete, duplicated, fragmented, and missing.

### Identification of molluscan and other lophotrochozoan pNPs

To date there is no publicly available program or script to perform a direct identification of pNPs and prohormones in transcriptomic or proteomic datasets. To circumvent this limitation, a pipeline comprising several distinct bioinformatic strategies was implemented and executed based on previous works^10,16^. All the major steps are described in detail below (Fig. 6B).

### Identification of signal peptide cleavage sites and establishment of the secretome databases

The initial identification of potential new lophotrochozoan pNPs and prohormones was started with the identification of the signal peptide cleavage site using the program signalP 4.0^82^. The program was executed under the following parameters: −m −n −u 0.45 −U 0.50, in which the parameters −m and −n control the output files (i.e. fasta file with the mature protein sequence and a gff annotation file, respectively) and the parameters −u and −U define the cut-off scores used to predict and identify the signal peptide cleavage site. The protein sequences that failed to present a signal peptide cleavage site were discarded. All subsequent analyses were carried out using the mature protein sequences (i.e. the protein sequence without the N-terminal signal peptide) (Fig. 6B).

### Removal of known folded protein domains, search for repetitive motifs, and establishment of the neuropeptidome databases

To avoid false positive results two distinct approaches were implemented: (1) identification and exclusion of sequences with known folded protein domains using the program hmmsearch;^83^ (2) the identification of repetitive motifs (cleavage sites) using a local Perl script. The similarity searches using hmmsearch were executed using the mature lophotrochozoan protein sequences as queries and the PFAM-A and B database under default parameters and a defined e-value of 1e-10. The protein sequences without matches to the PFAM-A or B database were screened for repetitive cleavage sites motifs using the following Perl regular expressions: (R|K)*GKR(R|K)*, (R|K)*GRK(R|K)*, (R|K)*GRR(R|K)*, (R|K)*GKK(R|K)*, (R|K)*KR(R|K)*, (R|K)*RK(R|K)*, (R|K)*RR(R|K)*, (R|K)*KK(R|K)*, (R|K)*GR(R|K)*, (R|K)*GK(R|K)*. All mature lophotrochozoan proteins with a known folded protein domain and/or lacking any of the aforementioned repetitive motifs were discarded (Fig. 6B). Redundancy was removed from the neuropeptidomes using cd-hit^84^ with the parameter −c defined as 0.95 (sequence identity threshold).

### Similarity searches against a curated non-redundant dataset of 6,692 pNPs

To avoid unrelated spurious sequences, to optimise the subsequent analyses, and to decrease computational burden in the phylogenetic steps, similarity searches were carried out using the blastp alignment tool^85^. The predicted neuropeptidomes were used as BLAST queries against a well-curated database composed of 6,692 metazoan pNPs^10,16,27,28^ using a loose e-value of 1e-03. The protein sequences without any similarity against the pNP database were removed from the next step of the pipeline (Fig. 6B).

### Clustering, multiple sequence alignment, motif identification, and illustration of the biological sequences

The remaining lophotrochozoan pNPs and peptide prohormones (i.e. full length proteins including signal peptide) were used as input for the program CLANS^54^, a Java application for visualising protein families based on pairwise similarity, together with the curated dataset of 6,692. The input dataset was clustered during approximately 20,000 rounds using local psi-blast using the following parameters: −evalue 1e-06 matrix BLOSUM62 -num_iterations 3 (Fig. 1A). Metazoan peptides that failed to connect to any molluscan peptides were excluded from the map. The large and strongly connected cluster composed by repetitive peptide sequences at the center of the map (Fig. 1B) was re-analysed using CLANS and a non-iterative blastp similarity tool with an evalue of 1e-06. To help the identification of the peptide families, clusters were identified with the function “find cluster: convex clustering” under the default parameters. To improve and aid the overall classification and phyletic distribution of the pNP and hormone families in each cluster, motif searches using meme^86^, multiple sequence alignments using mafft^87^, and additional phylogenetic inferences using mrbayes^88^ were employed. The diagram of the proteins was drawn using IBS software^89^. Any isolated pNP cluster smaller than 3 sequences and without any recognisable conserved domain(s) were excluded from the map. The peptide families identified in molluscan and lophotrochozoan representatives were classified according to their evolutionary origins following criteria established by Conzelmann *et al*.^16^ to distinguish pNP/prohormone families present in the last common ancestor (LCA) of eumetazoans, bilaterians, protostomians, and lophotrochozoans. Additionally, peptides with their evolutionary origins tracing back to the LCA of Mollusca and different class-level taxa were also identified and classified. The final 3D maps were collapsed to 2D after the clustering for easier visualisation (Fig. 6C, Supplementary Datasets S2 and S3).

### Phylogenetic analysis

Multiple sequence alignment files for each family were generated with the program mafft under the following parameters–maxiterate 1000 –localpair. The trimming of the poorly aligned regions in order to increase the accuracy of the subsequent phylogenetic inferences was performed with trimal or BMGE^90,91^. Phylogenetic analyses were performed with mrbayes using the appropriate best-fit model of amino acid substitution as determined by Akaike information criterion (AIC) implemented in prottest3^92^. The number of generations used in each phylogenetic run was determined using a convergence diagnostic (i.e. the standard deviation of split frequencies). All the runs were performed using the samplefreq parameter defined as 1000 and a relative burn-in of 25%. The final phylogenetic consensus tree was edited with Figtree (http://tree.bio.ed.ac.uk/software/figtree).

## Supplementary information

Supplementary Information

Supplementary Dataset S1

Supplementary Dataset S2

Supplementary Dataset S3

Supplementary Dataset S4

Supplementary Dataset S5

## Data Availability

All data generated and/or analysed during this study are included in this published article (and its Supplementary Information Files).
