# Supplementary material for: Extensive conservation of the proneuropeptide and peptide prohormone complement in mollusks
Source: Sci Rep. 2019 Mar 19;9:4846. doi: 10.1038/s41598-019-40949-0 (PMC6425005; doi:10.1038/s41598-019-40949-0)
Supplement: Supplementary file 1 — Supplementary Information [file 41598_2019_40949_MOESM1_ESM.pdf]

Supplementary information: Extensive conservation of the proneuropeptide and peptide prohormone complement in mollusks

De Oliveira[1], AL; Calcino[1], A and Wanninger[1] A.*

[1]Department of Integrative Zoology, Faculty of Life Sciences, University of Vienna, Althanstraße 14, Vienna, 1090, Austria

ORCID ID:
Andreas Wanninger: 0000-0002-3266-5838
André Luiz de Oliveira: 0000-0003-3542-4439
Andrew Calcino: 0000-0002-3956-1273

*author for correspondence: andreas.wanninger@univie.ac.at

Authors'email:
André Luiz de Oliveira: andre.luiz.de.oliveira@univie.ac.at
Andrew Calcino: andrew.calcino@univie.ac.at
Andreas Wanninger: andreas.wanninger@univie.ac.at

Supplementary information

BUSCO assessment of gene content and completeness of the lophotrochozoan protein sets (Supp. Fig S1) 3

Manually curated catalog of molluscan pNP and peptide hormone (Supp. note S1) 5

Trimmed multiple sequence alignments and phylogenetic analysis of four groups of prokineticin-like peptides in the monoplacophoran *Laevipilina hyaline* (Supp. Fig. S2) 5

Structure of molluscan and lophotrochozoan ELH/DH44 peptides (Supp. note S2) 7

Structure of molluscan and lophotrochozoan myomodulin and PRXamide peptides (Supp. note S3) 12

Structure of molluscan and lophotrochozoan FCAP peptides and identified repetitive peptide motifs (Supp. note S4) 23

BUSCO assessment of gene content and completeness of the lophotrochozoan protein sets (Supp. Fig S1)

Supplementary Figure S1: BUSCO assessment of gene content and completeness of the lophotrochozoan protein sets. Gray stars indicate species in which genomic data were available. Datasets were classified into BUSCO metrics as follows: C: complete (C) and single copy (S); complete (C) and duplicated (D); fragmented (F); and missing (M). Black stars correspond to predicted protein sets obtained from available genomic data.

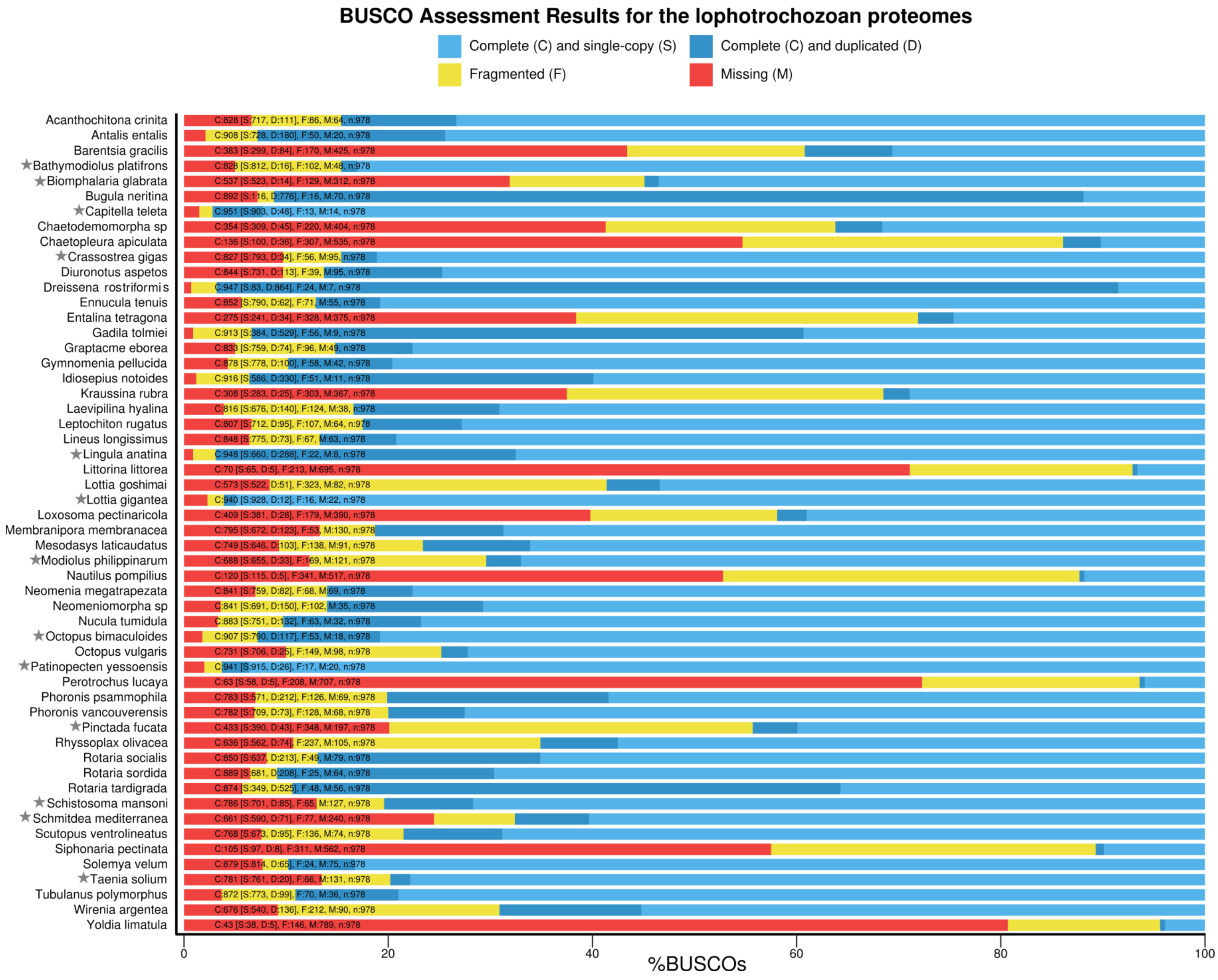
BUSCO Assessment Results for the lophotrochozoan proteomes
Complete (C) and single-copy (S)
Complete (C) and duplicated (D)
Fragmented (F)
Missing (M)
Acanthochitona crinita
C:828 [S:717, D:111], F:86, M:64, n:978
Antalis entalis
C:908 [S:728, D:180], F:50, M:20, n:978
Barentsia gracilis
C:383 [S:299, D:84], F:170, M:425, n:978
★Bathymodiolus platifrons
C:828 [S:812, D:16], F:102, M:48, n:978
★Biomphalaria glabrata
C:537 [S:523, D:14], F:129, M:312, n:978
Bugula neritina
C:892 [S:116, D:776], F:16, M:70, n:978
★Capitella teleta
C:951 [S:903, D:48], F:13, M:14, n:978
Chaetodemomorpha sp
C:354 [S:309, D:45], F:220, M:404, n:978
Chaetopleura apiculata
C:136 [S:100, D:36], F:307, M:535, n:978
★Crassostrea gigas
C:827 [S:793, D:34], F:56, M:95, n:978
Diuronotus aspetos
C:844 [S:731, D:113], F:39, M:95, n:978
Dreissena rostriformis
C:947 [S:83, D:864], F:24, M:7, n:978
Ennucula tenuis
C:852 [S:790, D:62], F:71, M:55, n:978
Entalina tetragona
C:275 [S:241, D:34], F:328, M:375, n:978
Gadila tolmiei
C:913 [S:384, D:529], F:56, M:9, n:978
Graptacme eborea
C:833 [S:759, D:74], F:96, M:49, n:978
Gymnomenia pellucida
C:878 [S:778, D:100], F:58, M:42, n:978
Idiosepius notoides
C:916 [S:586, D:330], F:51, M:11, n:978
Kraussina rubra
C:308 [S:283, D:25], F:303, M:367, n:978
Laevipilina hyalina
C:816 [S:676, D:140], F:124, M:38, n:978
Leptochiton rugatus
C:807 [S:712, D:95], F:107, M:64, n:978
Lineus longissimus
C:848 [S:775, D:73], F:67, M:63, n:978
★Lingula anatina
C:948 [S:660, D:288], F:22, M:8, n:978
Littorina littorea
C:70 [S:65, D:5], F:213, M:695, n:978
Lottia goshimai
C:573 [S:522, D:51], F:323, M:82, n:978
★Lottia gigantea
C:940 [S:928, D:12], F:16, M:22, n:978
Loxosoma pectinaricola
C:409 [S:381, D:28], F:179, M:390, n:978
Membranipora membranacea
C:795 [S:672, D:123], F:53, M:130, n:978
Mesodasys laticaudatus
C:749 [S:646, D:103], F:138, M:91, n:978
★Modiolus philippinarum
C:688 [S:655, D:33], F:169, M:121, n:978
Nautilus pompilius
C:120 [S:115, D:5], F:341, M:517, n:978
Neomenia megatrapezata
C:841 [S:759, D:82], F:68, M:69, n:978
Neomeniomorpha sp
C:841 [S:691, D:150], F:102, M:35, n:978
Nucula tumidula
C:883 [S:751, D:132], F:63, M:32, n:978
★Octopus bimaculoides
C:907 [S:790, D:117], F:53, M:18, n:978
Octopus vulgaris
C:731 [S:706, D:25], F:149, M:98, n:978
★Patinopecten yessoensis
C:941 [S:915, D:26], F:17, M:20, n:978
Perotrochus lucaya
C:63 [S:58, D:5], F:208, M:707, n:978
Phoronis psammophila
C:783 [S:571, D:212], F:126, M:69, n:978
Phoronis vancouverensis
C:782 [S:709, D:73], F:128, M:68, n:978
★Pinctada fucata
C:433 [S:390, D:43], F:348, M:197, n:978
Rhyssoplax olivacea
C:636 [S:562, D:74], F:237, M:105, n:978
Rotaria socialis
C:850 [S:637, D:213], F:49, M:79, n:978
Rotaria sordida
C:889 [S:681, D:208], F:25, M:64, n:978
Rotaria tardigrada
C:874 [S:349, D:525], F:48, M:56, n:978
★Schistosoma mansoni
C:786 [S:701, D:85], F:65, M:127, n:978
★Schmitdea mediterranea
C:661 [S:590, D:71], F:77, M:240, n:978
Scutopus ventrolineatus
C:768 [S:673, D:95], F:136, M:74, n:978
Siphonaria pectinata
C:105 [S:97, D:8], F:311, M:562, n:978
Solemya velum
C:879 [S:814, D:65], F:24, M:75, n:978
★Taenia solium
C:781 [S:761, D:20], F:66, M:131, n:978
Tubulanus polymorphus
C:872 [S:773, D:99], F:70, M:36, n:978
Wirenia argentea
C:676 [S:540, D:136], F:212, M:90, n:978
Yoldia limatula
C:43 [S:38, D:5], F:146, M:789, n:978
0
20
40
60
80
100
%BUSCOs

Manually curated catalog of molluscan pNP and peptide hormone (Supp. note S1)

Supplementary note S1: Manually curated catalog of molluscan/lophotrochozoan peptide families containing trimmed multiple sequence alignments and peptide logos. Multiple sequence alignments were generated using the program mafft, and subsequently trimmed with trimal program. Peptide logos were generated using meme software. To access the high-quality pdf files please copy in your browser the following address: https://phaidra.univie.ac.at/view/o:924430.

Trimmed multiple sequence alignments and phylogenetic analysis of four groups of prokineticin-like peptides in the monoplacophoran *Laevipilina hyaline* (Supp. Fig. S2)

Supplementary Figure S1: Trimmed multiple sequence alignments and phylogenetic analysis of four groups of prokineticin-like peptides in the monoplacophoran *Laevipilina hyalina*. The branch support values are posterior probabilities values. Multiple sequence alignments were generated using the program mafft, and subsequently trimmed with trimal program. The phylogenetic inferences were performed with mrbayes. For detailed information see “Material and methods – “Phylogenetic analysis”.

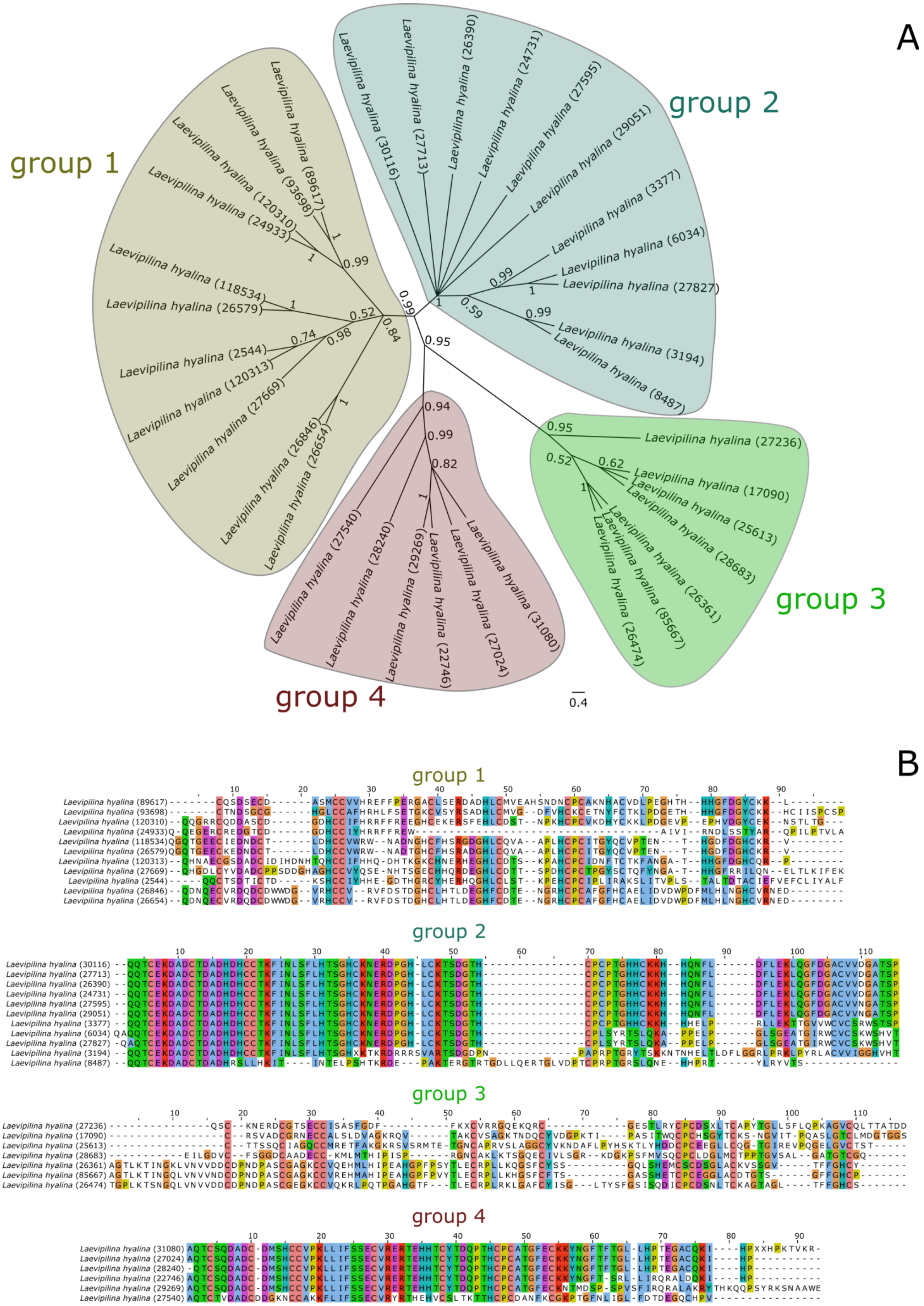
A
group 1
group 2
group 3
group 4
Laevipilina hyalina (89617)
Laevipilina hyalina (93698)
Laevipilina hyalina (120310)
Laevipilina hyalina (24933)
Laevipilina hyalina (118534)
Laevipilina hyalina (26579)
Laevipilina hyalina (2544)
Laevipilina hyalina (120313)
Laevipilina hyalina (27669)
Laevipilina hyalina (26846)
Laevipilina hyalina (26654)
Laevipilina hyalina (30116)
Laevipilina hyalina (27713)
Laevipilina hyalina (26390)
Laevipilina hyalina (24731)
Laevipilina hyalina (27595)
Laevipilina hyalina (29051)
Laevipilina hyalina (3377)
Laevipilina hyalina (6034)
Laevipilina hyalina (27827)
Laevipilina hyalina (3194)
Laevipilina hyalina (8487)
Laevipilina hyalina (27236)
Laevipilina hyalina (17090)
Laevipilina hyalina (25613)
Laevipilina hyalina (28683)
Laevipilina hyalina (26361)
Laevipilina hyalina (85667)
Laevipilina hyalina (26474)
Laevipilina hyalina (27540)
Laevipilina hyalina (28240)
Laevipilina hyalina (29269)
Laevipilina hyalina (22746)
Laevipilina hyalina (27024)
Laevipilina hyalina (31080)
0.4
B
group 1
group 2
group 3
group 4

Structure of molluscan and lophotrochozoan ELH/DH44 peptides (Supp. note S2)

Supplementary note S2: Structure of molluscan and lophotrochozoan ELH/DH44 peptides, highlighting the identified repetitive peptide motifs. Motif identification was performed with meme software, and the presence of signal peptide was revealed with signalP program.

Molluscan DH44

>*Crassostrea gigas* (405974916) MolluscaBivalvia
MKMKLNVMAVIVTALFVSVDAFILQDQDNQPDLSEADSIPVE**KR**GRLSLTADLRSLARMLE
AHRKRFIASRFPYDSIRKKLFRYG**KR**SPVPETLEYNEGEDRSNALDSEFPSLKVEEESPIY
NI**KR**QRLSVNGALSSLADMLAANGRQRMMSEMAMNRQRLFGLG**K**

>*Patinopecten yessoensis* (PY_T23501) MolluscaBivalvia
MKIQNNHLLLVSLVMIYCTAVNTYALDKTASDILLEQHHSGNSGVNVPFE**KR**AIPLSLNGD
LRMLARMLYASQRRRRVDRFASVRQQMSNLG**KR**SGGVQHDKDNDDPIRNSQDTSIRTE
QQTQIRSTVPDYRLVSADGYQNVHPRSSLAYEDPEEFQ**KR**SQRLSINGALSSLADMLAA
SGRRQLKEELAVNRQRLLKLG**R**

>*Pintada fucata* (29971.t1) MolluscaBivalvia
MFTSFFQLDKNAIKKVGTFISPIR**KR**ELTENNYEYMSNMRERAIYILAIMVVLSNIGGTVSE
NDDSGSSEEKPAFIEIEDSPEVS**KR**TYISLNGDMRSLAKMLMRHYGNRSVKRPVENYTSL
RKKLYALG**KR**DAKPTLRDLLKDWIFKQT**KR**QRLSVNSALASLADMVSADGHRRMKEEMS
SNHQRLLGLG**KR**

>*Acanthochitona crinita* (21836) MolluscaPolyplacophora
FLCLCVLMLAARGQSQSPLYPITLDDNIKPTTDRETRENWPLDSDSAHLTQ**KR**LGQLSLN
HDLKTLARLLENQQVRRTSDARQKLRIIG**KR**GHIMSTYFPLFLDGFYQKFQQGPMSVRGG
KRGNGGPRLSVSGALSTIADMVQAQNRRRMFHEMAQNRASLSGIG**KR**SNTPTQSLPAP
VRGMSNIDNGRDYK

>*Gadila tolmiei* (32365) MolluscaScaphopoda
MVTSQLAITVIVLTASCVIATREVSKGVNGFFGYSDLDDFLKTHSDDNIAKDIQKDVAITTN
DESEPSVK**KR**SPDRLKERRLWLLRLLKSRMALRRRVPKYQPA**KR**QRLSINGALSSLADM

LEAQGRQRYNNEMSYTHQRLLALGKRGGIFAKSGNNIPHVDGLSDDVMTPENSKGGEL
NENQKGHWIPIKYNDDKATMEKGNDAAQEEEQKQWNNNKRARFSINGPLSSLVDMLDN
RERDMVANRLRALGRR

>*Antalis entalis* (110445) MolluscaScaphopoda

MPLNSLTIVTFAAAVLVVCGSMEQIHRYDENKDISTEYEKRGPHLSINNNLRSLALLLKASD
QRRAANDIHSHLRSLGKRGGGNYVMHMLWNMLRTRQKEGHQKRQRYSPPKRQRLSIN
GALSTLADMLEAQGRHDYNNEMSMTRQRLLELGKRDIPSDTKRDDQFQDAIEQLPDKRA
RLSVNGPLSSLVDLLNSNDRSEVTNRLLALGKRGEPEQ

>*Graptacme eborea* (30760) MolluscaScaphopoda

MPLNSLRIAICAATVLVVFGSLEQIHNYEADDMPREYDKRLPKLSINNNLRSLANMLKARD
ARRAANKIHSHLRSLGKRGSHHFINMLIRLLQSRRNYSRKPPRYSPSKRQRLSINGALSTL
ADMLEAQGRHDYNNEMSMTRQRLLELGKRTMSAAIKADDEFLDGSEQDLMPNKRARLS
VNGPLSSLVDLMNSNDKSEVTNRLL

Gastropoda ELH

>*Aplysia parvula* (ELH1) MolluscaGastropoda

MGRPSYRSAAAISLLMCLILSALCASSESAVVHGSSFATERAVKSSPYLVLSPADEMVGM
STANEAFDKSPSYYDDDDDDFVKNEKRRLRFHKRRIRFNKRPQEVSGLKPVMMSRASA
SADENSLFDLYNTDGAMYQRDLRAPRLRFYSLRKRAAGDEDKSEENNPETESHSRRKR
SALTPSIRSLRSSLESGIAKRISINQDLKAIADMLIVEQKQEREKYLADLRQRLLNKGKRSS
EVALAASDKGDEERELLNTLSNLLE

>*Aplysia parvula* (B26147) MolluscaGastropoda

AISLLMCLILSALCASSESAVVHGDDFAAERAVKSSPYLVLSPADEMVGMSNNEKRRLRF
HKRRIRFHKRPQEVSGLKPVMMSRASASADENSLFDLYNTDGAMYQRELRAPRLRFYSL
RKRAAGDEDKAEEHNPETESHSRRKRSALTPSIRSLRSSLESGIAKRISINQDLKAIADMLI
VEQKQEREKYLADLRQRLLNKGKRSSEVALAASDKGDEERELLNTLSNLLE

>*Aplysia parvula* (AAA27766) MolluscaGastropoda

CNISPNVPHPLRSLRQFRVSSRSWRRLRRRKSSKILALPGPLPCRRNGRHVNNEKRRLR
FHKRRIRFHKRPQEVSGLKPVMMSRASASADENSLFDLYNTDGAMYQRELRAPRLRFYS
LRKRAAGDEDKAEEHNPETESHSRRKRSALTPSIRSLRSSLESGIAKISINQDLKAIADMLI

VEQKQEREKYLADLRQRLLNKG**KR**SSEVALAASDKGDEERELLNTLSNLLE

>*Aplysia parvula* (P17686) MolluscaGastropoda

AISLLMCLILSALCASSESAVVHGDDFAAERAVKSSPYLVLSPADEMVGMSTANEAFDKS
PSYYDDDDDDFVNNE**KR**RLRFH**KR**RIRFH**KR**PQEVSGLKPVMMSRASASADENSLFDL
YNTDGAMYQRELRAPRLRFYSLR**KR**AAGDEDKAEEHNPETESHSRR**KR**SALTPSIRSLR
SSLESGIA**K**ISINQDLKAIADMLIVEQKQEREKYLADLRQRLLNKG**KR**SSEVALAASDKGD
EERELLNTLSNLLE

>*Aplysia dactylomela* (ACO95731) MolluscaGastropoda

LR**KR**AAGETEQSEGQNPETESHSRR**KR**SVLTPSLSSLGESLESGIS**KR**ISINQDLKAITDM
LLEEKIRERQRYLADLRQRLLQKG**R**KSSDVSLLTSNKGDEERELLKT

>*Lymnaea stagnalis* (P06308) MolluscaGastropoda

MKMSGLLSKPDYGVVGIVFTVVFCCWCSSSTTHALSIAEPGRDRYD**KR**SPTGHGVEVVE
SGEDYGSNRPQPVYGDEDEEDSADVYVGSDESSSGEKTRLTAA**KR**RLRFN**KR**RLRAS**K**
RRLRFH**KR**RVDSADESNDDGFDRKAREPRLRFHDVR**KR**SATAEEGSENAEIEESHLGNS
RSRRSAGSAPSSANEVQRS**KR**LSITNDLRAIADSYLYDQHKLRERQEENLRRRFLELG**KR**
GSAFFDHIPIIFGEPQYDYQPFK

>*Aplysia californica* (AAB84096) MolluscaGastropoda

MKANTMFIILCLTLSTLCVSSQFTPVLGKIFVTNRAVKSSSYEKYPFDLSKEDGAQPYFMT
PRLRFYPIG**KR**AAGGMEQSEGQNPETKSHSWRERSVLTPSLLSLGESLESGIS**KR**ISINQ
DLKAITDMLLTEQIQARRRCLAALRQRLLDLG**KR**DSDVSLFNGDLLPNGRCS

>*Aplysia californica* (AAA27742) MolluscaGastropoda

MKANTMFIILCLSLSTLCVSSQSTSVHGKIFVPNRAVKLSSDGNYPFDLSKEDGAQPYFMT
PRLRFYPIG**KR**AAGEMEQSEGQNPETKSHSWR**KR**SVLTPSLSSLGESLESGIS**KR**ISINQ
DLKAITDMLLTEQIQARRRCLDALRQRLLDLG**KR**DSDVSLFNGDLLPNGRPQLKTISNLLD

>*Aplysia californica* (AAA27748) MolluscaGastropoda

MKRPNNRPTNTMSLILCLTLSSHNGEDVFSNE**KR**RLRFH**KR**RLRFDRRDQDEGNFRRIP
TNAVSMSADENSPFDLSNEEGAVYQRDLRAPRLRFYSLR**KR**AAGEMEQSEGQNPETES
HSRR**KR**SVLTPSLSSLGESLESGIS**KR**ISINQDLKAITDMLLTEQIRERQRYLADLRPRLLE
KG**KR**SSGVSLLTSNKDEEQRELLKAISNLLD

>*Aplysia* sp. (AAA27747) MolluscaGastropoda

MKRPNNRPTNTMSLILCLTLSSLCVSSQSASVHGKNFATNRAVKSSSPFVVLSPDDNVVS
MSGENGYRSALREAFDKSSRDYDDNGEDVFSNE**KR**RLRFH**KR**RLRFDRRDQDEGNFR
RFPTNAVSMSADENSPFDLSNEDGAVYQRDLRAPRLRFYSLR**KR**AAGGMEQSEGQNPE
TESHSRR**KR**SVLTPSLSSLGESLESGIS**KR**ISINQDLKAITDMLLTEQIRERQRYLADLRQR
LLEKG**KR**SSGVSLLTSNKDEEQRELLKAISNLLD

>*Aplysia californica* (AAA27746) MolluscaGastropoda

MFFGAGFLLFARRQHSCGERMLYDDLRLSLVFPSQIRQTCNLFYFSLSQIEHISRDLVSVK
SSNSFYRQRFHSPQNRHFQQAKAYVM**KR**PNNRPTNTMSLILCLTLSSLCVSSQSASVHG
KNFATNRAVKSSSPFVVLSPDDNVVSMSGENGYRSALREAFDKSSRDYDDNGEDVFSN
E**KR**RLRFH**KR**RLRFDRRDQDEGNFRRFPTNAVSMSADENSPFDLSNEDGAVYQRDLRA
PRLRFYSLR**KR**AAGGMEQSEGQNPETESHSRR**KR**SVLTPSLSSLGESLESGIS**KR**ISINQ
DLKAITDMLLTEQIRERQRYLADLRQRLLEKG**KR**SSGVSLLTSNKDEEQRELLKAISNLLD

>*Lottia gigantea* (XP_009066138) MolluscaGastropoda

MTSYAMPRSYSQVPEELSSQDQSRDYYEPLNAILHRLRELILMQPHSLRETIRYTQITPTT
PIPIAMK**KR**SRLSINQELKSLANLLVLRENKRREAQKTKLRSKLLSIG**KR**SMPQNQDRDHV
IEETPVSDRDMFLTELFAEISEQEMTYVLGQVLGSEIAAKGGNEYQGQERLPYEI

>*Charonia tritonis* (AQS80499) MolluscaGastropoda

MTTMAFLLPILAAFFFLGLSSVQGLPTSKASHVTSGVAPERGLHKVTARSSGVKALT**KR**DI
SLNQDLKSLANMLLAREYDRILSNRMNREFLRKIG**KR**GSSSLVGGFEDVMDLLPGEPQE
DSLPSWWKCSDCDEENKFGRNSAPSSPPPSPRNRWGLAGSP

>*Biomphalaria glabrata* (XP_013067835) MolluscaGastropoda

MILFISLSTATLVLRSPEAAAWTDIAAINCEFRDERRQKATPKLRFN**KR**YRLRTS**KR**RLRF
QKKKQPYGDDSEYGRPEDSGSAYERDLRGPRLRFHAHDVR**KR**STYWGDAAEDLDREQ
FRQRRSSPLARLV**KR**VPIGNDLLALADLLFIERQKQAYSALKAMMDEAG**KR**

Lophotrochozoan DH44

>*Platynereis dumerilii* (DH44-2) Annelida

MPTPLIQGLLSVCVLIALISLSLALNDDSAEFESSEGLH**KR**AVKLSVNNGLSALANSMKDKE

LEDGIQTSHAQLFRMGKRPSFSFNQDLKSLADAHGMHSAENSRARLLNLGKRVMFSVN
GGLQSLADSMNEGPSHGNLMALGKRPAFSVNHGLDTLAQAMGSDGSSQSKLLSLGKRP
AFSVNHGLDTLAQAMGSEGSSSQSKLLSLGKRPAFSVNHGLDTLAQAMGSEGSSSQSK
LLSLGKRPAFSVNHGLDTLAQAMGSEGSSSQSKLLSLGKRPAFSVNHGLDTLAQAMGSE
GSSSQSKLLSLGKRMQLSFNQGLQSLADSMSNDGGRSRLMNLGKRGQLSINNELQSLA
HSFKPNGDSHMSLLNLGKRAPLSVNAELHSLAHSFRPNSQASLLDLGKRAPLSINAELHS
LAHSFNGDGEGRARLLNLGKRVPLSINAELQSLAHSYQPNSQMRLMSLAKRVPLSVNQG
LVSLASSMRGDRAEASRNFLQSLGKRAPLSIGQEMQSLSDAYNSHSGNPLLNLGKRMAL
SVNHELRTLGDAMRHGQAAPSLLSLGKRFDNQNDHEHVRIMKRSPGARRLSINSELWSL
AQMARDARSHEDRKNAQALLDHLGKRSV

>*Platynereis dumerilii* (DH44-1) Annelida

MPSTPRLGLLTLCIYLAIFTLTIATNDDRPDSSESDLDIHKRTRKLSVNTGLDALADSLRDR
DSMEDVKHSQSQLFRLGKRPAFSVNQDLSSLADAYGHGNAEESRARLLSLGKRVPLSV
NSGLQSLADSMSDSSQNRLLALGKRPAFSVNHGLDTLAEAMGMNGHNRLMSLGKRVPL
SINHGLQSLADSMADDDRQSRLQMLGKRGPLSINNELHSLADAYGNQDSPLLQLGKRGP
LSVNAELHSLASSFAPGSHASLLNLGKRMPFSVNAELQSLAHSFKDGDSQSQLLRLGKR
APLSVGAELQSLADSYDGGDSRSRLLSLGKRIPFSVNQDMQSLADSMNGNRGSESNSFL
QSLGKRAPLSIGQEMQSLSDAHMSGYNPLLNLGKRVAFSVNQELQTLGDAMRQGDDRL
LQLGKRSAKSHEANSRSKRVPGARRFAFNSELWSLAEMARHSRSQQERKDVQDLLDNL
GKRSV

>*Tubulanus polymorphus* (27387) Nemertea

MHSKHGSMITLTTVFILAVIVAIVEGKPLSDEYYEPLPSEFKTNLPSENSMREKDNDLYRKV
AQIAKMVLNRGKGDMSEYKYDDDSVDKRGGGSLSIVGSLDALTDMLSAQQARKFQNQL
KANKMRLMRLGKRDVDSKFRH

>*Lineus longissimus* (24664) Nemertea

MFRHLPFTIGVAIVLLSCLEARPYAIQEEYLPLDDEFEADAFRENGDEKRSMDLSVSGSLA
ALSDMLMWQRQQQQQQLNALGKRGSEFNNQETQELLKQLQLLRDSASGMRKRSGSE
LSVMGSLDALSDIISSQNARHKHDMMQANHQRLLRLGKRDTSANLEKTKNEETKKAD

Structure of molluscan and lophotrochozoan myomodulin and PRXamide peptides (Supp. note S3)

Supplementary note S3: Structure of molluscan and lophotrochozoan myomodulin (highlighted in yellow) and PRXamide (highlighted in light blue) peptides and identified repetitive peptide motifs. Motif identification was performed with meme software, and the presence of signal peptide was revealed with signalP program.

Gastropoda myomodulin -1

>*Theba pisana* Myomodulin-1-like2 Mollusca Gastropoda
MQISCVISLAFVVSLQFTVGFSDDQADSKASTDTALSRA**KR**ESYNMLRLG**R**GLNMLRLG**K**
RNDESTAQDEDDLEDMLAWRAGYYEPYTLDSYEHAYPEDDIEVPAHGRF**RR**STPSSKA
GLSSEVAQQLESSPKDSIPADLKLNAEEDQFENFPDDNVNFYDDFADGIIQPTEEGDEQD
KRSLGMLRLG**KR**QLSMLRLG**KR**SLGMLRLG**KR**EPEDDEY**KR**SLGMLRLG**KR**QLSMLRL
G**KR**ALGMLRLG**KR**EDENFDDIDSEDG**KR**SMSMLRLG**KR**PMSMLRLG**KR**PMSMLRLG**K**
RPMSMLRLG**KR**EDDE**KR**SLGMLRLG**KR**STQ

>*Theba pisana* Myomodulin-1-like1 Mollusca Gastropoda
MQISCVISLAFVVSLQFTVGFSDDQADSKASTDTALSRA**KR**ESYNMLRLG**R**GLNMLRLG**K**
RNDESTAQDEDDLEDMLAWRAGYYEPYTLDSYEHAYPEDDIEVPAHGRF**RR**STPSSKA
GLSSEVAQQLESSPKDSIPADLKLNAEEDQFENFPDDNVNFYDDFADGIIQPTEEGDEQD
KRSLGMLRLG**KR**QLSMLRLG**KR**SLGMLRLG**KR**EPEDDEY**KR**SLGMLRLG**KR**QLSMLRL
G**KR**ALGMLRLG**KR**EDENFDDIDSEDG**KR**SMSMLRLG**KR**PMSMLRLG**KR**PMSMLRLG**K**
RPMSMLRLG**KR**PMSMLRLG**KR**PMSMLRLG**KR**EDDE**KR**SLGMLRLG**KR**STQ

>*Aplysia californica* NP_001191423.1 myomodulin Mollusca Gastropoda
MQVYMLLPLAVFASLTYQGACEETAAAQTSSDASTSSASSEHAENELSRA**KR**GSYRMM
RLG**R**GLHMLRLG**KR**GGPVEPESEENLETLLNLLQGYYSDVPEYPSEFDDTDLAYPYEEY
DAPAHPRY**RR**STPPTDGVVAPDVLQKGSSEFEDFGDSQLDESDEGYYGYDPENYLYGD
FEDYLEPEEGGLGEE**KR**SLSMLRLG**KR**GLSMLRLG**KR**EGEEGDEMD**KK**QDESLNDDFE
NDDI**KR**TLSMLRLG**KR**PMSMLRLG**KR**PMSMLRLG**KR**PMSMLRLG**KR**PMSMLRLG**KR**P
MSMLRLG**KR**PMSMLRLG**KR**PMSMLRLG**KR**PMSMLRLG**KR**PMSMLRLG**KR**PMSMLRL
G**KR**DDDEKE**KK**SLNMSRLG**KR**STQ

>*Perotrochus lucaya* (22260) MolluscaGastropoda 4412 329 119 38

MKYILFSRAAFFLLLVLVNEGSSEEKSESNAEEQTLSRERRGLNMLRLGRGLQMLRLGGL
NMLRLGRSSPPLSTEELTNLLYNLIENEKLLADSEDDDSYTGEDYMYPSGRFRREIPDSE
QNTKGKDIDEFESEYESEQEVPQDGTDTYLVDEDDLGNYEKRGLVMLRLGKRPMNMLR
LGKRPMNMLRLGKRPMNMLRLGKRPMNMLRLGKRPMNMLRLGKRPMNMLRLGKRPM
NMLRLGKREDGDQTEKRGLSMLRLGKRPDDSTGEPKQ

>*Aplysia californica* NP_001191423 myomodulin neuropeptides 1 precursor

MQVYMLLPLAVFASLTYQGACEETAAAQTSSDASTSSASSEHAENELSRAKRGSYRMM
RLGRGLHMLRLGKRGGPVEPESEENLETLLNLLQGYYSDVPEYPSEFDDTDLAYPYEEY
DAPAHPRYRRSTPPTDGVVAPDVLQKGSSEFEDFGDSQLDESDEGYYGYDPENYLYGD
FEDYLEPEEGGLGEEKRSLSMLRLGKRGLSMLRLGKREGEEGDEMDKKQDESLNDDFE
NDDIKRTLSMLRLGKRPMSMLRLGKRPMSMLRLGKRPMSMLRLGKRPMSMLRLGKRP
MSMLRLGKRPMSMLRLGKRPMSMLRLGKRPMSMLRLGKRPMSMLRLGKRPMSMLRL
GKRDDDEKEKKSLNMSRLGKRSTQ

>*Lymnaea stagnalis* GastropodaMollusca

MQGAFLITFIVLTMTLVSIGNTEESGGQTSSNDKTEPAQSRTKRRSREMGRVVRGLQML
RLGKRDSVSTSEDPGDIDDILSLLQAYQEQNPEFAFNEEERELSGDELEVPEHHRFRRST
ENSGVAPQEVQQSQSFKDSGEHELKLEEAEPYLYFPDGDFYYGDVDELLEGDNEDGSA
DKRQIPMLRLGKRSMSMLRLGKREEDDADFDEEKRSLSMLRLGKREDDNFEDSFDEEN
DKRSLSMLRLGKRPMSMLRLGKRPMSMLRLGKRPMSMLRLGKRPMSMLRLGKRPMS
MLRLGKRPMSMLRLGKRPMSMLRLGKRPMSMLRLGKRPMSMLRLGKREDDEEKRSLA
MS

>*Lottia gigantea* jgi|Lotgi1|187713|estExt_Genewise1.C_sca_200247 myomodulin precursor-like

MLRLGRGLQMLRMDLQKITEGGEEEMKQQEAENYPEIEGYPVFADDVEYKNEDEKRPM
NMLRLGKRPMNMLRLGKRPMSMLRMGKRPMSMLRMGKRPMSMLRMGKRPMSMLRM
GKRPMSMLRMGKRPMSMLRMGKRPMSMLRMGKRPMSMLRMGKRPMSMLRMGKKS
DEDHSIETRKINMLRLGR

>*Lottia* sp. Myomodulin 1 FL499954 Mollusca Gastropoda

MFSVNFPLLLVSCLFLADLVYSEEEVSDTKTHDDSLSRTKRAGLNMLRLGRGLQMLRMG
KRADFPMLRMGRSEPQYTTDEDDAIRQLIIDILTQQAADRDYFYPESYPYPPPQPRYRRS
VSEFPQKEIDMQDLQKITEGGEEEMKQQEAENYPEIEGYPVFADDVEYKNEDEKRPMNM
LRLGKRPMNMLRLGKRPMNMLRLGKRPMNMLRLGKRPMNMLRLGKRPMNMLRLGKR
PMNMLRLGKRPMNMLRLGKRPMNMLRLGKRPMNMLRLGKRPMNMLRLGKKSDEDHSI
ETRKINMLRLGRSD

>*Deroceras reticulatum* ARS01383.1 myomodulin 1 MolluscaGastropoda
MHRRGLISLAVAVCLQLSLGYADNDASKANSDSSQDASLSRAKRGGYDMLRLGRGLNM
LRLGKRMYDSTSDLNENTPSNWADLQDYVEHDPENVGFDYSTFPESLEDSIIQAQEGKY
GKDLVDMGKRKMSMLRLGKRSVDNFDEDASAHFRQRRSASSVGGAVASEVLQSTNQA
SKDLEPVDLKDEDDDELLVEYPNDISQSEEDIDGKEWLPVISMGKRQLSMLRLGKRSLGM
LRLGKRESEDDEEKRALGMLRLGKRQLSMLRLGKRSLGMLRLGKRPSDDLDEVYGDED
DLTSEDGKRAMSMLRLGKRPMSMLRLGKRDDEEKRDMSKRSAL

Cephalopoda myomodulin-1

>*Octopus vulgaris* (18621) MolluscaCephalopoda
LLCKHVHLTATIRFCELVLCVLFPRGNCNEDTNPSQSKSKLNENNVAEQHLDKRSSGEYN
PHDLKTIVAAILERQEQGQKQSSALRSMNELAGDQYSGDALSRVVQLLRRSMPSDFDEF
ERRLAPVPRLGRLKKRSVLSNPNKEVDYDENEANDNNESADNILFPRQITLPRYGKDEGS
DAADAEQYLSSDCEIFDAYGTCVQYKIPRENVKRQVRMLRLGKRQMHPIKIDDETGMDK
RSEDFNVDGNSGESKRAVSMLRLGRSFNAGGESEENEIPVFDEAKRAVSMLRLGRSGP
FLDKRALSMLRLGRSEFDQENTALPIMYPGPNGFESNKRAVSMLRLGRSMSADDKRAVS
MLRLGRSGFDTMKRAVSMLRLGRNSGYPSEKRAVSMLRLGRSGSDEDKRAVSMLRLG
RSGSDEDKRAVSMLRLGRSGADIEDEKRAVSMLRLGRGGADNMKRAVSMLRLGRSGS
DDMNTKRAVSMLRLGRSGNDNVGEDKRAVSMLRLGRSDSNANNKRAMAMLRLGRSND
TSAKET

Bivalvia myomodulin-2

>*Crassostrea gigas* gi|405963046|gb|EKC28655.1| Myomodulin neuropeptide 2 MolluscaBivalvia
MFLSLISGSADSIDESHSRVRRGGLSMLRLGGLQMLRLGKRGMPMLRLGRSNGLSETDE
DFMYPDESELDEGRRQVPLPRYGKDLQQQLQLEWLQSVLDSDLENNGVRIIRPAGRPG

RFRRSLKEDNGEEKDSEERRHIPHPRIGRLIQLDDLKYPTSNLGSYYLTDSNVYTDKRGMPMLRLGRGMPMLRLGKRLQADDSKRGMPMLRLGKRTNVQADSASQQETNQNKRGMPMLRLGRNAN

>*Solemya velum* (31137) MolluscaBivalvia

MRCVLTAVVVLCLNHYVTGSLDDVSNADEDDSPLSRVRRGGLSMLRLGRGLQMLRLGKRGIPMMRLGRSTSDMYSPEELRYLLAALLSDEKSMSERQLPLPRYGKDLDWQTFLEGVLRGEEASEVLRRSRPALAILESDDARPIRPAPRPGRYRRSLQTMKPDNLDQSNKRAVPIPRIGREREDERDRAVPVPRFGRCLEYDRAAPLPRIGRFQDYDYPTMYDDGEKRGMHMLRLGRAMNMLRLGKRPMNMLRLGRSDVDSDSLSEAEKKALHLLRLGKRPVNMLRLGRDGQDEKRAVNSENDANEATNEDQLTHNVDDRSVKMLRLGRSAENSRTN

>*Ennucula tenuis* (43649) MolluscaBivalvia

MKYLIPFTIVLLYYQTASGSTADDTSLEEDENSQLSRVRRGGLSMLRLGRGLQMLRLGKRGLPMMRLGRSTDSQFSPQELQYLLGALLSDSKSLGYRQVPLPRYGKDLDLQSLLGGLSPEDATEILRRSRPLVGYWNPEGSTGRQIRPAPRPGRFRRDVPDVPMSPDQDEDIQNDDKEEYHEGRAVPIPRIGREDEYRAVPIPRIGRFENYDYYPMSEEEKRGMNMLRLGRGMQMLRLGKRPMSMLRLGRAGDQE

>*Patinopecten yessoensis* (PY_T18874) MolluscaBivalvia

MKYILPIILLLHCHQLITGSTDNDNTSEENGSPLNRVRRGGLSMLRLGRGLQMLRLGKRSMPMNRIGRSLDTLSSDELKYLLVSVLGDKFNHRRQVPLPRYGREDREDAELQWLLEHIGSDRVNTDESSEGLYDLDDESPRQIRLAPRPGRFRRSTDEQDKQDATQKGAYIQDVEEDKNEEEKAIPLPRVGRILYGGERALPLPRLGRDEMYDYVYTLEPAKDSEDSDVDKRGMHMLRLGRGMNMLRLGKRPMSMLRLGRSEIQQEANKGDDKRSLSMLRLGKRLRMLRLGKRPDDGDRSLRMMRLGKKDVDDSGSDSEHSVETRGMHMLRLGRNVYK

>*Pinctada fucata* scaffold1943.1 : 19966-31846 Mollusca Bivalvia

WIILLYNTGAEDEVLETNRQNAPLSRVRRGGLSMLRLGRGLQMLRLGKRAMPMLRLGRGASDSITQEQIRYIISLLLQEENYDNEPFRRQIPFPRYGKDLSSMQELIEELQRMPSQERRTDMYDLDEDSPRQIRPGPRPGRFRRSTAHMLQPDVSQEEAEVNSVERAPPLPRIGREQEDGEGEEGDIKVPMVKGNSDYYIDVAKDGSYYVADKRGMPMLRLGRGMPMLRLGKRPFKMLRLGRGSEGSDDEKRGMPMLRLGKRPFKMLRLGKRLESESESDKRAMAMLRLGRNSX

>*Dreissena rostriformis* (21149) MolluscaBivalvia

MLYGTPVALLLLSIYIVSGNEEVANDDMSASAATGESYDRVRRSGLSMLRLGRGLQMLRL
GKRALPMLRLGRSNPNGITDEDIQYLLSIVRQDRQVPLPRYGKDISKDLAFQYMLMNALK
NVEDNSNEYFDNEGSDSVSYAFPYESTSERQIRPAPRPGYRYSADGASTATNSNDDKDI
LSSDDSYKLDGLTDDKDKYSRVAPLMRYGKLAVDMPEYDEEIEKRNAMRMLRLGRGMR
MLRLGKRPSYSDSEEFSEADKRGALRLLRLGKRSGFRMLRLGRGTTDAEAENRAEENE
S

>*Dreissena rostriformis* (410984) MolluscaBivalvia

MLYRTPVALLLLSIYIVAGNEKVANDDMSASVATGESYDRVRRSGLSMLRLGRGLQMLRL
GKRALPMLRLGRSNPNGITDDDIQYLLSIVRQDRQVPLPRYGKDISKDLAFQYMLMNALK
NAEENSNEYFDNDGFDNVAYAFPYESTSERQIRPAPRPGYRYRRSVDGESSGTNSNDD
KDILSSDNSYKLDGLSDDKDKYSRVAPLMRYGKLAVDMPEYDEEIEKRNAMRMLRLGRG
LRMLRLGKRPSYSESEEFSEADKRGALRLLRLGKRSGFRMLRLGRGPTDPEGENRALGL
LGLMEKTEENES

>*Mytilus galloprovincialis* FL499954 MolluscaBivalvia

MKYSFTLLVVLYSYVVAGSSKVTESSHDDGQLNRVRRGGLSMLRLGRGLQMLRLGKRA
MPMLRLGRGVESYTPEEIRVIINTLIGEERGDRQVPLPRYGKDVEVQMLLQRLLGDPQFQ
ERRSMSLYDADDDSPRLINPGPRPGKYRYHRSLPDVPPQNVYDEEKFEDIN

>*Pinctada maxima* GT282875 MolluscaBivalvia

MMKYLVPLILLLHCYHKVTGAEDEVLETNRQNAPLSRVRRGGLSMLRLGRGLQMLRLGK
RAMPMLRLGRGATDSYTPEEIRYIISLLLREENYDNEPFRRQIPFPRYGKDLEWQTLLEEL
QNQMPSQERRTDIYDLDEDSPRQIRPGPRPGRFRSTPMLHPDISQYEKEKRK

>*Pinctada maxima* gi|262326550|gb|GT282875.1|GT282875 Adult silver lipped oyster Mollusca Bivalvia

MWICGFLCFVVVLNFRMMKYLVPLILLLHCYHKVTGAEDEVLETNRQNAPLSRVRRGGLS
MLRLGRGLQMLRLGKRAMPMLRLGRGATDSYTPEEIRYIISLLLREENYDNEPFRRQIPFP
RYGKDLEWQTLLEELQNQMPSQERRTDIYDLDEDSPRQIRPGPRPGRFXRSTPMLHPDI
SQYEKEKRK

Scaphopoda myomodulin-2

>*Antalis entalis* (136710) MolluscaScaphopoda

MKNIFYAILAIVFFYVQNGTTEETDAKLTSEQDDHALSRVRRGGLSMMRLGRGLHMLRLG
KRSLPFVRLGRSDLLDGVDSDELDRIIYELLFRYNQDFNDRQIPMPRYGRKRDGSDGNE
DEGYEYVPTSGLSPPPRLGRFKRSTPDSDMEKRAVPVPRFGRYLEGIRAVPIPRFGRYLY
DYDGLAKRNLGMMRLGRAGLTTLRLGRRALPMMRLGKRPLNTLRLGKREDDSQTEVDK
RLLNVLRLGKREIAEEQSSEKVADKAE

>*Graptacme eborea* (17073) MolluscaScaphopoda
MMKNIFYAILAIVVFYVQNGITEDADVKLAANEEDHALSRVRRGGLSMLRLGRGLQMLRL
GKRALPMLRLGRSSPSEDGVPSDLDLLVYALLYNYDKDFRLRQVPIPRYGRGEEGDDDT
DGEGYDYVSPNQIRPPPRLGRFKRSALSSDEDTENKRAVPVPRLGRFLGYRAVPVPRIG
RYIFGDDMYDKRSLGMLRLGRAGLHTLRLGRRALPMLRLGKRPLNTLRLGKREDDSQQ
NEVDKRLLNVLRLGKREALQADSSEKDAQQAD

>*Gadila tolmiei* (27403) MolluscaScaphopoda
MKHLLCTLLAIIVLYVQNGSSTETDADESNGEPLSRFRRGGLSMLRLGRGLHMLRLGKRS
LSMLRLGRRSSPETPAPSDLDILLYELISKLKYGKNLDERQVPVPRYGRQSDADDEELGG
GMSYDLDYNRQARGPPPRLGRFRRSADNNKEVEMDKERAVVPEPRRVQMEDIPAGNW
NSEYGSDFNDYEAEKRTMNMLRLGKRRLNMLRLGKRDFADQEMDKRMLNMLRLGKRE
APNVNIAGNDKALSPEK

>*Gadila tolmiei* (48009) MolluscaScaphopoda
MIKLRMKHLFCTLLAIIILYVQRGSSTVSNSEGSNTVPLSRYRRGGLSMLRLGRGLHMLRL
GKRSLSMLRLGRRSSPEAPSPSDLDFLLYGLLRKLKYGKALEDRQVPVPRYGRQEEGED
MDDDVMPYGYEPVRGPPSGLNRFRRSADSDEEMKNDDQDNERAVPAPRLGRDEFASN
WNADYGYGNGEYEAAKRSLSMLRLGKRRLNMLRLGKREFEEKQEAEKRMLNMLRLGK
REAPNSDLTEHENSISS

>*Gadila tolmiei* (104924) MolluscaScaphopoda
CFSTSILVMIATIYFMAIYLANCQSSDISNDEIENDHNNIFGDMANNDDLDGNNDEDGDISS
LAMPSVHYSNKRSLRLMRLGKKKRGIRLMRFKKHQATEKLYPNEATADGYATLYNNNKK
RALRLMRLGKRLIGNHKRALRLMRLGKRSRNYYSSPLVTFRNNADTAS

>*Antalis entalis* (201982) MolluscaScaphopoda
MKNIXYAILAIVVFYVQSGTTEETDAKLTADKEDHALSRVRRGGLSMMRLGRGLHMLRLG
KRSLPIMRLGRSSPSENGGPSDLDLLVYDLLYNYVQGFPLTDRHLSHATGREG

Gastropoda myomodulin-2

>*Lottia* sp. Myomodulin 2 FC775836 MolluscaGastropoda

MNGLSHIIPVFLLNLGLTCLLCNGAPTEDSLSRVRRGGWSMLRLGRGLQMLRLGKRTHP
SGLDIYLDRDLHERQVPLPRYGKDLDWQNFLERHMLGNTEMDEEKRESNLPLTDPFLSE
AIDGYPHMRPAPRGGRFKRSAGRFRYYPEVRSEERAVALPRFGRLIEKELKHKDGNDES
KERAVPAPRFGRNPH

>*Deroceras reticulatum* ARS01384.1 myomodulin 2

MNSLYETCIYFCAIAFIAGTSLVQAKVESSRAETSANKGQFSAARLGRGLQMLRLGKRSV
NLDSLNPDQANSHLTSNDVQAVLASIFDQPRDESRRQPPLPRYGRDSNNNVKGRLLDDA
MTDNSGVYQADFFPLSSQRFFFRPAPRGGRYRKSVPAGRLAYGSYISQDSVDRARALAF
PRFDQFIEELSHLQPKAVPRPRIGRYQNDQDTNSFQAKLV

>*Aplysia californica* NP_001191658 myomodulin neuropeptides MolluscaGastropoda

MWKILETCSCFLVVAVLSGLGKAQPESFSGSAVTDDSTSGANKRGWSMLRLGGLQMLR
LGKRGGSLDALRSGHQVPMLRAGRGSPDTSGRLDANELYAVLSAILDEPRDQSRRQPPL
PRYGRDNNGVARDLLDALASDGESSSNFDLLSSLNNGPSYFRPAPRGGRYKRSLPDAG
PADYPSLEDYLVQSRQFARPYSSRAVALPRIGRFSGSPRLQAKAVPRPRIGRQESQMRE
AKSAE

>*Aplysia californica* ABA70768 myomodulin gene 2 neuropeptide precursor MolluscaGastropoda

MWKILETCSCFLVVAVLSGLGKAQPESFSGSAVTDDSTSGANKRGWSMLRLGRGLQML
RLGKRGGSLDALRSGHQVPMLRAGRGSPDTSGRLDANELYAVLSAILDEPRDQSRRQP
PLPRYGRDNNGVARDLLDALASDGESSSNFDLLSSLNNGPSYFRPAPRGGRYKRSLPDA
GPADYPSLEDYLVQSRQFARPYSSRAVALPRIGRFSGSPRLQAKAVPRPRIGRQESQMR
EAKSAE

>*Lymnaea stagnalis* CN810524 Mollusca

MKNFIQATFVIFALATFTDQISAQAEPFLEEDGSDDTSTDVNKRVWGKLRQGNGLQMLRT
SGHPMLRLSRSSSDSDILKSLSPSDLQELLALLVDEPRDDMRRQPPLPRYGRDSSSVRR

SDNSAGDNRLFRFLASLGGKSSSRPAPRGGRYRRSVVDDKASYGDFLTPNATVRPLKPS
PCPDMEDRIX

>*Haliotis asinina* GT275969 MolluscaGastropoda

KRPMNMLRLGKRPMNMLRVGKRPMNMLRLGKRPMNMLRLGKRPMNMLRLGKREDET
EGEEKRALGMLRLGKRSDEKVDGDAGVSTQQ

Cephalopoda myomodulin-2

>*Idiosepius notoides* 205418 MolluscaCephalopoda

MNINPAFVCILLCLQLNEGICETQETHKNDIENESTPVQRERRAVGMLRLGRGLQMLRLG
KRAPYEDLKELVATMLDRQAPLPRYGKEEDLLDEAYPADALPQLLRRSHPLYLEGDEEA
YGQEAPIPRLGLLHKRSAETRHAPLPRYGKEPDYEDLNNDDFSDEENEEPSLEDDGESG
VGSQIYERQPPLPRYGKDEIAGFDCDTKDQNGNCLGYGDIEKKDVRMLRMGRQVNMLR
MGKRGLGMLRMGRNPGNLKRAVSMLRLGRSDNSENTKRALAMLRLGR

Polyplacophora myomodulin-2

>*Chaetopleura apiculata* (43386) MolluscaPolyplacophora

MKFWGSLIAFFICLHQHYGNXAEIEENAAQQSDSPMVRATRGGLGMLRLGRGVGFRTRG
VRMLRLGKRSDPETAK

>*Leptochiton rugatus* (2356) MolluscaPolyplacophora

MRIFGTLIAFFLCLHQHYGSSAEIEENAAQHNDGPMVRATRGGLGMLRLGRSMNFRTRG
VRMLRLGKRSDYNSPLNHLTYDDLKDILYSLYDEEKHPMARQPPLPRYGRELDWQDDVD
SSSEDSGIDKKLSPGLAMQLLAADAYGSHMRPAPRLGRFRRSLPDAPPSSESTEEKDNE
ESKERAVPMPRFGRLIDEIEKDEEDDEKDSGERAVPMPRIGRENEEYILENEMYPENEKR
ALQMLRLGRSMNMLRLGKRPEAYDTTDDKRSLALLRLGKRRMRMLRLGKREPENGDEA
DKRSLKLLRLGKRMDDHAIDTRALSMLRLGRSQDTNDKRSLKLLRLG

>*Acanthochitona crinita* (66817) MolluscaPolyplacophora

MKLWGTIIAFCIGLHHHCGHSAEVAENVQQENNSPMVRATRAGLNMLRLGRSLGFRTRG
VRMLRLGKRADPLDNLTYDDLKELVYSLTSEEKHPHERQPPLPRYGKRMSPDQAIQLLE
SDSYGSHMRPAPRLGRFKRSALDDAESPIDSAADDVDRTKDALPIPRFGRLTDENDSVE
NEKAVDEKAIQENEDELDTYKRGLPMLRLGRSMKMLRLGKRDSLGEDDKRSLALLRLGK

RGMRMLRLGKREPELDKRALRILRLGKREEDDHPIETRALRLLRLGRSQQDDKRSLKLLR
LG

>*Rhyssoplax olivacea* (10027) MolluscaPolyplacophora

MKLCGTLIAFFICLHQHYGNSAEIEENAAQQSDSPMVRSTRGGLNMLRLGRAGKYRTRG
VRMLRLGKRADPLENLTYDDLKDILSSLYDAEKHPMVRQPPLPRYGRELDLLDDYDPMA
KRMSPDAALQLLEAEAYGSHMRPAPRLGRFKRSPDSSENEIASDVIEDGSDTVRALPIPR
FGRLVEGKGKSADDEKDGDERAVPMPRIGRAKVQENGLESDKRGLAMLRLGRSMKMLR
LGKREPVAFEEP

Aplacophora myomodulin-2

>*Scutopus ventrolineatus* (20023) MolluscaChaetodermomorpha

MHSMKTTTSGLLFFILCLLQLRGAFSEEQDATVSREKRGLSMLRLGRSGRIHSRGIRMLR
LGKRDSADNKLTPEELKDLILSLLENENRQYERQAPIPRYGKDLSWQYEKRPFQDPLSLY
GVDFGYGLRPVPRLGRLKRDVNDAEKQSEDGEGKERAVPMPRIGRENEDEDGSDAKID
RYEGDERAVPMPRIGRLIDAEEDDYPTLYDLADKRGIGMLRLGRGMRMLRLGKRIPEDY
KRSLALLRLGKRRMSMLRLGKREGEDDDMSSDKRSMRLLRLGKRRGVSMLRLGRSNT
ADNTPEDKRSLKLLRLG

>*Wirenia argentea* (45237) MolluscaNeomeniomorpha

MNTLAAAFGILCALHVIQGITGEQDEKRRTLGMLRLGRNYNRGIRGFRMLRLGKRSDVDS
GLRKLLFSLAESKNFDERQAPLPRYGRELGYDDGADSLPIDGELYAPLRPVPRLGRFRRS
VDLENDEKDEYTKAVPMPRIGKDTDEVSERGLNIPRFGREPDLYYDMPEVNRRAMHLLR
LGKRRMKMLRLGKRGRGMTMLRLGRSSDDQVDKKSS

>*Gymnomenia pellucida* (42352) MolluscaNeomeniomorpha

MNAIAAVFGVLCAINALHGVTGETDEKRRSLGMFRLGRGYNRGTRGFRLLRLGKRSDVD
NGLQKLLFSLAESKDFTQRQAPFPRYGRELSYDEIGSSLPFGDDLYAPIRPVPRLGRFRR
SADIEDEEEKSEFTKAVPMPRIGKDLDGLYDPEMADAYYAMPEVNRRSLHLLRLGKRRM
KLLRLGKRGRGMYMLRLGRSSDKQETPAS

>*Neomeniomorpha* sp. (40249) MolluscaNeomeniomorpha

MKSVLTIFGALCILQILQGYTVRANDEDDSAIDKRARSLRGMNMLRLGRSGNSMSRFIRG
FHMLRLGKRSDVENSLRKLLYSIAENKDYTRQAPLPRYGREDMDLPLDIYAPVAPVPRLG
RFKRSIQEDRAVPMPRIGREEEENE

Lophotrochozoan myomodulin-PRXamide

>*Lingula anatina* (g16064.t1) Brachiopoda

MHVLATATICLLFQAFHGYLADGFERDTRGLGIMRMGKRANQLKMIRLGRGMRMLRLGK
RGDPYMYGYPYDLFYEPRNDIAEQFRRQIPTYPRIGKDLDYAPFSEEWNWPAANPRPRV
GKELQDYEWAAAEEANHDASLPFVLSRKKRSAVDRPESHYDEKKGSPSLPRLGEPTAK
RSDDEPLPYLPNEDYEQYVRGIIPYPRIGRYEAYDMPPVYDEDPYFDTYADKRAFRGLRL
GKRMRMLRLGSDSQKTNDLSKEDSDFSQHEEKRKMRMLRLGKRSENSKISKVAPAKNE
IASKN

>*Lineus longissimus* (36186) Nemertea

MPSPVVKLTLGVAILTFIQGVCLGTDSDDTSMRERRAVPMLRMGRSLGMIRMGKRDLRM
LRMGRSYPDGEDLIGRQVTYPRVGKDLESEEVLRSNPSLPRLGKDLGGIPVYNGLLLDQL
LKEVHNHQRYARSVKEENSNHVSEEDSKERAIPFPRLGKDLTELEEGDRAIPYPRIGKDL
NELEAYKRAMGMIRMGKKAMGMIRMGKREEEEKRAMGMIRMGKKAMGMIRMGRGQE
DPSPEEKRAMGMIRMGRSQIDDVTDKRAMGMIRMGKRDDEMDKKSLRLVRMGRAMG
MIRMGKRDDEEAGSDELSKRAMHLIRMGRKRAMGMIRMGRASSDEGTDEEKRAMHLIR
MGKRSASETAQNAKQ

>*Tubulanus polymorphus* (22219) Nemertea

MLPCKVQIAIILTTVFCFSDITCTKEDDSNAHERQRRSMPLVRMGRAFGLVRMGKRSDMK
LIRMGRSYTDDDTAEELYSRQPHFPRIGKDLDSVDEEVRSPNFPRLGKDVYRQLLIRDLL
DDLDQRPAYGRFTRSARTARSAPGPDATQAHNEDDHSHERAIPFPRLGREEDENESKTD
EKDQRDRAIPFPRLGRDEDELNELKRAFELVRMGKRNLDESPDMNLLRPARAFSLVRMG
KRSSADDVKRAFSLVRMGKRSEDEDEKRAFKMFRMGKRAFSLVRMGKRSEDDKRAFG
LVRMGKRSEDEKRAFGLVRMGKRSEDEKRAFGLVRMGKRDEEDSKRAFSLVRMGKRN
EEDSKRAFGLVRMGKRDEAAKRAFGLVRMG

>*Platynereis dumerilii* myomodulin_mRNA1_Annelida

MTNCFALCLLVSCLCALSSAENDREKRAIRMLRMGKRGFGMLRLGRSAPYDQYESRQLF
DVPRMGKDLVDIMEPSKRHPPIPRVGNLDKVIDEYRRMVADEATSFDELPRFGRFVRSA
EEVANEVEGSHKEKREVSGGVLPRLGLRDVENEIRAAPLPRLGYRAIPRPRVGYRDLETL
PRLGLRELEYQRAAPLPRLGLRENFADEDDKEERAVPLPRLGLRDLDKKAVSMLRMGRS
EETDTEHEMDKRAMSMLRMGKRGMSMLRMGKREEEIEPEVDEKRAMGMLRMGRAMS
MLRMGKKDAIEQYKRKMSMLRMGKRGMSMLRMGKRDDEQQEFTEEGKRKMNMLRM
GKRAMSMLRMGKRDLDEYREQKRAMGMLRMGKREFNDSPDEKRAMGMLRMGKRDS
EDELQEKRAMGMLRMGKREYENLDEKRAMGML

>*Capitella teleta* CapteP226517 Annelida

MKVALLFSLQCIYVIASADDSARARRDLSMLRMGKRSEFSSLPPLVPPSYDLSADDFDER
QVWMKIPRVGKDIDEDTPHLRLARYPPVPRLGSALDSLIEEYRRDIGSNDDEDEIRQVLFK
IPRVGRNRRSADDQENVEMKEQVKRAAPLPRLGMLEERAAPLPRLGLYERAAFLPRLGY
RDLDEEERAAPLPRLGVREEDYENEVGDNSYLKEEDERAAPLPRLGYYEKRNVGMLRM
GKRPMSMLRMGKRPMSMLRMGKRPMSMLRMGKRPMSMLRMGRSMDEAQPEQQKR
AMSMLRMGKRGMNLLRMGRSEQPAEEEEKRAMSMLRMGRSEPAVEAEKRPMSMLRM
GKRPMSMLRMGKREMDDEVIPVVEAEKRPMNMLRMGKRDTDDNQPIVEEDQQAQSS

>*Helobdella robusta* Annelida Lophotrochozoa myomodulin

MEKMAMFFLMATLSVMSGIVHCSDHSKSVSPELGEQGHDANLLRILEEPQQEDSNKRAI
GMLRMGRSLPYYTDDDAGMKRAVSYLRMGRSGLNDKKAVNMLRMGKRVVSLLRMGR
SSSANNNQLDEQYDEEANSPSRKFFYDSPNYFVDDAEKENYKLAHDNKRAVSMLRMG

>*Schmidtea mediterranea* Platyhelminthes

MSKLTYFIFMIMLFIFVQTIDINSNQYEEDYDPNDDHELDKRAYRLMRMGKRAVRLMRMG
KKAVRLMRLGKRSDMA

>*Schmidtea mediterranea* Smed-mpl-2 Platyhelminthes

MQYLFAAFIFMAYYIRCEDFNENIIYPELFEDSEQYNAIPWNKRAVRLMRLGKRIAPLKRA
VKLMRLGKREE

Structure of molluscan and lophotrochozoan FCAP peptides and identified repetitive peptide motifs (Supp. note S4)

Supplementary note S4: Predicted molluscan and rotiferan FCAP proneuropeptide sequences. The predicted signal peptides are highlighted in green, predicted mono-, di-, and tribasic cleavage sites are shown in bold red, the predicted bioactive 13 amino acid length FCAP copies are underlined and highlighted in yellow, and finally predicted bioactive FCAPs with variable length are underlined and highlighted in light blue.

Aplacophora FCAPs

>*Scutopus ventrolineatus* (26893) MolluscaChaetodermomorpha

MMKLLAAAVFCVGVVLRVSSSAPAGNAAEDNAGD**KR**NAPPAQGLESDLKPVGEN**KR**YFDSLGGFQVPGA**KRR**GFDSLGGFEVPAY**KR**HFDSLGGFHIPGV**KR**PYFDSLGGFQIPVS**RKR**HFDSLGGFEVPYS**KR**SWYFHSSPEFPVPGSH**KR**YFDSLGGFEVQDT**KR**SPYFDSLGGFQVPAQ**KRR**YFDSLGGFHVPGN**KR**AGYFDSLGGMQLLQ**KK**SGDAKSHNSEMG**R**A**KR**GFDTLGGFQLLPS**KR**NFDTLGSFQVPYN**KRR**FDNLGGWHILPG**KR**EHDNE**KK**YFDSLGGFEVQQ

>*Gymnomenia pellucida* (45208) MolluscaNeomeniomorpha

MLTRIVTLFLVVSGVISASSKNVHSGKVSI**KKR**GLDSLGGSALMPH**KK**GLDTLGHSALMPE**KK**GFDTLGGSAFMPD**KRR**SD**KR**GLDSLGHSALMPH**RR**GFDSLGNSALMPD**KR**GFDSLGNSALMPD**KR**GFDSLGNSALMPD**KR**GFDSLGNSALMPD**KR**GFDSLGNSALMPY**KR**GFDSLGNSALMPD**KR**GFDSLGNSALMPD**KR**GFDSLGNSALMPD**KR**GFDSLGNSALMPY**KR**GFDSLGNSALMPD**KR**GFDSLGNSALMPD**KR**GFDSLGNSALMPD**KR**GFDSLGNSALMPD**KR**ALIPN**KR**VMFSRLRNF**RR**PYLQ**RR**GFDTLGGFQLLPS**K**

>*Wirenia argentea* (62011) MolluscaNeomeniomorpha

MILKLFTFCLVLSEAIGAPAKEEVHEGLHL**KK**KSLRSLPH**KK**GFDSLGNSAFMPD**KR**GFDSLGHSAFMPE**KK**GFDSLGNSAFMPD**KR**GFDSLGHSAFMPQ**KK**GFDSLGNSAFMPD**KKR**GFDSLGHSAFMPE**KK**GFDSLGNSAFMPD**KK**GFDSLGHSAFMPQ**KK**GFDSLGNSAFMPD**KR**GFDSLGHSAFMPE**KK**GFDSLGHSAFMPE**KK**GFDSLGNSAFMPD**KR**GFDSLGHSAFMPE**KK**GFDSLGNSAFMPD**KKR**GFDSLGHSAFMPE**KK**GFDSLGNSAFMPD**KR**GFDSLGHSAFMPE**KR**GFDSLGNSAFMPD**KR**GFDSLGHSAFMPQ**KK**GFDSLGNSAFMPD**KR**SMFSRFRNF**RR**PFLQ**RR**GFDTLGGFQLLPS**K**

Polyplacophora FCAPs

>*Acanthochitona crinita* (67303) MolluscaPolyplacophora
MTTLLFVLVLVIFSVGQPAPVENADSGNQNEIE**R**LPEESAKAVADIG**R**ILTALQQKHGDGT
NSFDGDVDTTND**KR**TFDSLGGFAIHPG**KR**YFDSLGGFAIHPG**KR**HFDSLGGFAIHPG**KR**
HFDSLGGFAIHPG**KR**HFDSLGGFAIHPG**KR**QFDSLGGFAIHPG**KR**HFDSLGGFAIHPG**KR**
HFDSLGGFAIHPG**KR**HFDSLGGFAVHPG**KR**QFDSLGGFAIHPG**KR**QFDSLGGFAIHPG**K**
RNFDSLGGLDVPY**KK**DNNEEYDAGNAVE**RR**ALDRLGGIDVPY**KK**TYFDSLGGFGIHPA**K**
RLAADNFLSGDNGSNELDN

>*Leptochiton rugatus* (8199) MolluscaPolyplacophora
MRQVLCWALIYVFLTVGHSAPVETVDDSNEEKGLHESKATLVTELG**R**LLELL**R**H**R**HENSL
DSGAYGSDYEDV**KR**TFDSLGGYAIHPS**KR**HFDSLGGFAIHPG**KR**HFDSLGGFAIHPG**KR**
HFDSLGGFAIHPG**KR**HFDSLGGYAIHPG**KR**HFDSLGGFAIHPG**KR**HFDSLGGFAIHPG**KR**
NFDSLGGFAIHPG**KR**HFDSLGGFAIHPG**KR**DSDNSDDIANPEI**KR**HFDSLGGFAIHPG**KR**
GTNTPDDLDQSYD**R**GYLSDDVAKTILDDDAGQSVYWPYNDVHPME**R**IES**R**SQEEKELL

>*Rhyssoplax Olivacea* (34683) MolluscaPolyplacophora
MMLCLTFLLTLSSTVLSASLEGVDTENQNEIQSLPGAKGKLVSDVGKLLTVL**R**E**R**QDDSE
RDYNGDAIDVPPEAS**KR**GFDTLGGIDVHGY**KR**QFDSLGGYAIHPG**KR**HFDSLGGFAIHP
G**KR**QFDSLGGYAIHPG**KR**QFDSLGGFAIHPG**KR**HFDSLGGFAIHPG**KR**QFDSLGGYSIH
PG**KR**HFDSLGGFAIHPG**KR**QFDSLGGYAIHPG

Conchifera FCAPs

>gi|266704586|gb|GT570646.1|GT570646 dlsmcb0_005726 Japanese scallop adductor muscle normalized *Mizuhopecten yessoensis* MolluscaBivalvia
LGGMWIHGY**KKR**GLDRLGGAYLHGF**KR**AMDNDGDNS**KR**SLDRLGGAYLHGF**KR**AMDN
DDVDS**KR**SLDRLGGAYLHGF**KR**AMDNDDVDS**KR**SLDRLGGAYLHGF**KR**AMDNGEVDS
KRSLDRLGGAYLHGF**KR**SMDNGEVDT**KR**SLDRLGGAY

>gi|223024814|gb|FL490716.1|FL490716 Mg_Nor01_50P13 Nor01 *Mytilus galloprovincialis* MolluscaBivalvia 3194 711 138

LEDTVNPNSAEELRMFLESNMEKNNEEKRGLDTLGGMSYLKRGLDTLGNMGFHSYKKK
GLDTLGNMGFHDFKKRGLDTLGNMGFHDFKKRGLDTLGNMGFHDFKKRSLDT

>*Nucula tumidula* (35751) MolluscaBivalvia

MRWCELGLAIIVVSSVLVKPTLQAPVAGPALQEKTDGTVENRSLAEDILEILKDNERNSDL
KSQSEKSWEQLYVPPVHSFPLDTLGGTIIHPARTLDLQSDIRDANIDSDSAIDGDGDYSGV
DKRAFDSLGGMQIHGYKRGLDSLGGMQIHGYKRGLDSLGGMQIHGYKRGLDSLGGMQI
HGYKRGLDSLGGMQIHGYKRGLDSLGGMQIHGYKRGLDSLGGMQIHGYKRGLDSLGG
MQIHGYKRGLDSLGGMQIHGYKRGLDSLGGMQIHGYKRGLDSLGGMQIHGYKRGFDSL
GGMQVHGIKRGLDSLGGMQIHGYKRSSAMKTDGSD

>*Crassostrea gigas* HG965451 MolluscaBivalvia

MKMHSVLYLGLLFSICYQTSAGDNKVQRYDPSEYNDLLQLLQGWTLTTPSASMTTLPPLD
NLGGAVVHKGYRESGDIEGDLYQQEPKSDNGVPENKVSSDSLDEDYFERFQDKYPSES
EFDKVSETYNDEDPSDDKEKRVLNSLGGFSLHGYKRGLSSLGGLNLHNFAKRGLNSLGG
MSLHDFKRGLSSLGGMSLHNFNKRGLNSLGGFGLHNYKRALSSLGGFSLHGYKRRLSS
LGGMSLHGGLNKRGLYRPGRMSLPGGFSKRGLSSLGGMSLHGGFSKRGLSSLGGMSL
HGGFSKRGLSSLGGMSLHGGFSKRGLSSLGGMSLHGGFSKRGLSSLGGMSLHGGFSK
RGLSSLGGMSLHGGFSKRGLSSLGGFRLHGYKRALSNLGGMNLYNFKRSLNDLEKNNN
SESPYDNLESSDIMSGTMDASKHKRSVPSEPRTDN

>*Patinopecten yessoensis* (PY_T21625) MolluscaBivalvia

MFTKFVIVTIFCWTTLVNCGPVKHDTPVSSTDNQPKRVQRSTPQEIHEQIMNELRGWTPS
PADTEVSSGHNNDPLDRLGGTYVHKGTRDADDAYDLLSVVEEDALPSEGDSTEDNVIPD
EEVSFGPSADKFNALRGFLQAIKEGNETPTKRSLDRLGGAFIHGYKRALDPLGGVYLHGY
KRSLDPLGGMWIHGYKKRGLDRLGGAYLHGFKRAMDNDDDNSKRSLDRLGGAYLHGF
KRAMDNDDVDSKRSLDRLGGAYLHGFKRAMDNDDVDSKRSLDRLGGAYLHGFKRAMD
NDDVDSKRSLDRLGGAYLHGFKRAMDNDDVDSKRSLDRLGGAYLHGFKRAMDNDDVD
SKRSLDRLGGAYLHGFKRAMDNDDVDSKRSLDRLGGAYLHGFKRAMDNDDVDSKRSL
DRLGGAYLHGFKRAMDNGEVDSKRSLDRLGGAYLHGFKRSMDNGEVDTKRSLDRLGG
AYLHGFKRSMDDGEVDSKRSLDRLGGAYLHAFKRAMVDDDDVDSKRSLDRLGGAYLH
GFKRAMDDDADLKRSLDRLGGAYLHGFKRSMDDDENEEKRSLDRLGGAYLHGFKRALD
PLGGAWLHNYKRALDPLGGAYLHGYKRALDPLGGAYLHQYKRSVDTDSQDKKDNSDPI
GEKQTVIVELDGWTRKTCKLVNDYREQTAREKLCCPGISTLNGDRLDDKVFAEIIF

>gi|163312613|gb|FC557999.1|FC557999 CAWC939.fwd CAWC *Lottia gigantea* from head (L) (AMQO01002371.1) Mollusca Gastropoda

MLSKVYMLCLEIFICCKISSILTSQLETKVTQKNEVRREKELYPLMNKKSEESFTISDYDDVDESAEKRYFDTLGGAFVHSFKRNSNPSKNKQFFEKLKNLLKVSKESSHKRALDDLGGINVHSFKRGLDDLGGVNVHGFKRGLDDLGGVNVHGFKRGLDDLGGVNVHGFKRSLDDLGGINVHGFKRGLDDLGGVNVHGFKRGLDDLGGVNVHGFKRGLDDLGGVNVHGFKRGLDDLGGVNVHGFKRGLDDLGGVNVHGFKRGLDDLGGVNVHGFKRGLDDLGGVNVHGFKRGLDDLGGVNVHGFKRGLDDLGGVNVHGFKRGLDDLGGVNVHGFKRSLDDLGGVNVHGFKRGLDDLGGVNVHGFKRGLDDLGGVNVHGFKRGLDDLGGVNVHGFKRGLDDLGGVNVHGFKRSFDGLGGMNVHGFKRGFGINVHRFKRGLDDLGGIHVHSFKRNFDDLGGMNIHGYKRALRDLNTIQADDFKRKLNSHGGMNVHGNKRGFDDLGGIDVHSFKREAGDLDETESNHVQHIENNASTNKR

>jgi|Lotgi1|116754|e_gw1.24.11.1 Feeding circuit activating peptides-like *Lottia gigantea* Lophotrochozoa MolluscaGastropoda

MNKKSEESFTISDYDDVDESAEKRYFDTLGGAFVHSFKRNSNPSKNKQFFEKLKNLLKVSKESSHKRALDDLGGINVHSFKRGLDDLGGVNVHGFKRGLDDLGGVNVHGFKRGLDDLGGVNVHGFKRSLDDLGGINVHGFKRGLDDLGGVNVHGFKRGLDDLGGVNVHGFKRGLDDLGGVNVHGFKRGLDDLGGVNVHGFKRGLDDLGGVNVHGFKRGLDDLGGVNVHGFKRGLDDLGGVNVHGFKRGLDDLGGVNVHGFKRGLDDLGGVNVHGFKRGLDDLGGVNVHGFKRSLDDLGGVNVHGFKRGLDDLGGVNVHGFKRGLDDLGGVNVHGFKRGLDDLGGVNVHGFKRGLDDLGGVNVHGFKRSFDGLGGMNVHGFKRGFGINVHRFKRGLDDLGGIHVHSFKRNFDDLGGMNIHGYKRKLNSHGGMNVHGNKRKLNSHGGMNVHGNKRGFDDLGGIDVHSFKREAGDLDETESNHVQHIENNASTNKR

>gi|325297025|ref|NP_001191518.1| feeding circuit activating peptides precursor [*Aplysia californica*] MolluscaGastropoda

MTFAASFRALLCVLFCAALVHCKTRTKRYVPHSELWRILAVVDELQREQAAEQRQEDALALALRSDIAGGGGGGQLADNVRWFPETYDYGALADRDVDKRVFDSLGGYEVHGFKKRGSLDAIPQDTDASSDKRALDSLGGFQVHGWKRALDTLGGFQVHGWKRGSGAEKRQVDRLGGFQVHGWKKRALDSLGGFQVHGWKKRGTGGQMHASSPRVVPWGSRSLLADTQSGHRWKRDTELVENRQTTGQQTEVNKRALDSLGGFQVHGWKRSGEAGKRQVDSLGGFQVHGWKRADDQGKRALDSLGGFQVHGWKRFDNSAGEKRALDSLGGFQVHGWKRAGDKKSLDSLGSFQVHGWKRFDNDISGQKRSLDSLGSFQVHGWKRSDQDNKRALDSLGGFQVHGWKRADDDGKRSLDSLGSFQVHGWKRADEDDKKSLDSLGSFQVHGWKRGDEDDK

RSLDSLGSFQVHGWKRADEDDKRSLDSLGSFQVHGWKRSDEDDKRSLDSLGSFQVHGWKRSDEDDKRSLDSLGSFQVHGWKRADEDDKRSLDSLGSFQVHGWKRNSPGLKRALDSLGGFQVHGWKRNNEYYSGAENEKRALDSLGGFQVHGWKRDQPGEKRSLDSLGSFQVHGWKRNLNNLGSFQVHGWKKNSADEMGDKPGVESYQDNSGKILSGKAQEFEGGDETGDIHGVVRTLSGVDASGKERENIKELDAKFKTNDGGVGVEHIFVDNVKSADDDVPSAGQM

>ARS01364.1 feeding circuit activating peptide 1 [*Deroceras reticulatum*] MolluscaGastropoda

MKHSGFQTRFLVIVCVCYATLVDAEMRSKRFSQNSAILKALTLENDLHHHQQQQQHNNYINSGTPFVSRYDPTSPDTPSDTEDPDEILAETVIPPSPWYSAIHSSVRYPLTHFYMGRQDEEKGKRFFDSLGGSEVHGFKRSAFGRLGSSPERFRENSDIIQMVPDTYDGALVDASRSWQGIDSVEKAEDANQQPLFEVHYLKPLIHKRALDSLGGFQVHGWKREAGKRSLSSLGGFQVHGWKRRQNEVAKKSLDSLGGFQVHGWKKRAVDAVDKLHMSKIESTASRITKSGGILSGVAEGKMAKLQESVDIDQSNGASGAVGKLVENKLVDGSTFGVNDSNLDRNSDYQYFGKRALDSLGDFQVHGWKRDEGKRALDSLGDFQVHGWKRDARAI

>*Gadila tolmiei* (26651) MolluscaScaphopoda

MVKLLNVQNTFRTLLCVLVFVSKSLTLATKDIKQGIPDNGKVYVVEPFDPDEGDTGLVIESGQISDGKESPLPVNDDSVGMSAQKKWLDTLGGWNVHGYKRSLDTLGGFNVHDYYKRTPNKRGRFSRLLRNNNNKRREYRSWLDTLGGLNVHGGKKRSLDTLGGFNIHDGGYKRQFDSLGGYNVHGGWKRALDTLGGFNIHDGYKRSFDTLGGFNIHDFKKRPFDTLGGFNIHGGYKRSEDKRQFDTLGGLNVHGGWKRAYMDGEGHEDEKRALDTLGGFNIHDGYKRALDTLGGFNIHDGYKRAFDTLGGFNIHDGGYKRAFDTLGGFNIHDGGYKRAFDTLGGFNIHDGKKRNSGDALEIEATRSKRDA

Rotifera FCAP-related

>*Rotaria tardigrada* (51758) Rotifera

MNTTNIPMMIVALVITTMCLLSANGEEEKKRTLDSLSGSDFFKKSLDSLSGNDFFKRDDEKRTLDSLSGSDFFKKSLDSLSGNDFFKRDEEKRSLDSLSGSDFFKKSLDSLSGNDFFKKSLDSLNGNDFFKKSLDSLSGNDFFKRSLDSLSGNDFFKRNIKAREHAVEELLRHSLYPHMSREHFRTLMHKQKFDDKDK

>*Rotaria tardigrada* (51760) Rotifera

MNTTNIPMMIVALVITTMCLLSANGEEEKKRTLDSLSGSDFFKKSLDSLSGNDFFKRDDE
KRTLDSLSGSDFFKKNLDSLSGNDFFKRDEEKRTLDSLSGSDFFKKSLDSLSGNDFFKK
SLDSLNGNDFFKKSLDSLNGNDFFKKNLDSLSGNDFFKRNTKAREHAIEELLHSLYPHML
NEHFRTRMHKQKVDDKDK

>*Rotaria tardigrada* (31473) Rotifera

MTAATMSTIFVALVIATMCQLGTMGEEEKKRTLDSLAGSDFFKKSLDSLSGNDFFKREQE
KRTLDSLSGSDFFKKSLDSLSGNDFFKREEEKRTLDSLAGSDFFKKSLDSLSGNDFFKK
SLDSLSGNDFFKKSLDSLSGNDFFKKSLDSLSGNDFFKRNMKPWRLHTISGRHSHHPLR
VYDLFSVINNQDK

>*Rotaria sordida* (17318) Rotifera

MTAATMSTIFIALVIATICQLGTMGEEEKKRTLDSLSGSDFFKKSLDSLSGNDFFKREDEK
RTLDSLSGSDFFKKSLDSLSGNDFFKREEERRTLDSLSGSDFFKKSLDSLSGNDFFKKSL
DSLSGNDFFKKSLDSLSGNDFFKRNMKPWGFNTILGRHSHHHHPSRVYDRFSVINHQDK

>*Rotaria socialis* (21748) Rotifera

MTTTNMSMMIVALVITTMCLLIANGEEGKKRTLDSLSGSDFFKKSLDSLSGNDFFKREDE
KRTLDSLSGSDFFKKSLDSLSGNDFFKRDDEKRTLDSLSGSDFFKKSLDSLSGNDFFKK
SLDSLSGNDFFKRSTKAREYAIAELLRHSLYPHMTREHFRTRFNKQKN
